# Research Progress of ODS FeCrAl Alloys–A Review of Composition Design

**DOI:** 10.3390/ma16186280

**Published:** 2023-09-19

**Authors:** Xi Wang, Xinpu Shen

**Affiliations:** 1 Key Laboratory for Anisotropy and Texture of Materials (Ministry of Education), School of Materials Science and Engineering, Northeastern University, Shenyang 110819, China; 2 Institute of Reservoir Engineering, College of Petroleum Engineering, China University of Petroleum (Huadong), Qingdao 266580, China

**Keywords:** ODS FeCrAl, accident-tolerant fuel cladding, oxidation resistance, irradiation, thermal stability, microstructure, mechanical properties

## Abstract

After the Fukushima nuclear accident, the development of new accident-tolerant fuel cladding materials has become a research hotspot around the world. Due to its outstanding corrosion resistance, radiation resistance, and creep properties at elevated temperatures, the oxide dispersion strengthened (ODS) FeCrAl alloy, as one of the most promising candidate materials for accident-tolerant fuel cladding, has been extensively studied during the past decade. Recent research on chemical composition design as well as its effects on the microstructure and mechanical properties has been reviewed in this paper. In particular, the reasonable/optimized content of Cr is explained from the aspects of oxidation resistance, radiation resistance, and thermal stability. The essential role of the Al element in oxidation resistance, high-temperature stability, and workability was reviewed in detail. The roles of oxide-forming elements, i.e., Y (Y_2_O_3_), Ti, and Zr, and the solid solution strengthening element, i.e., W, were discussed. Additionally, their reasonable contents were summarized. Typical types of oxide, i.e., Y–Ti–O, Y–Al–O, and Y–Zr–O, and their formation mechanisms were also discussed in this paper. All aspects mentioned above provide an important reference for understanding the effects of composition design parameters on the properties of nuclear-level ODS FeCrAl alloy.

## 1. Introduction

Since the beginning of the new century, with the continuous growth of global power demand and the dual challenges of energy shortage and environmental protection, countries all over the world have paid more attention to nuclear energy compared with fossil energy. As the most promising alternative to fossil energy, nuclear energy is considered a clean energy source with low carbon emissions and high efficiency. Nuclear power generation has a history of more than 60 years. However, due to the deep negative impact of nuclear accidents on human beings and the environment, the safety of nuclear energy has become the focus of attention around the world. For example, in the 1990s, after the accidents at the Three Mile Island and Chernobyl nuclear power plants, the United States, Europe, Japan, and other countries researched and tackled key issues actively for the development of the derived third generation reactors to prevent and mitigate serious accidents. Compared to the second-generation nuclear power units where the Fukushima nuclear accident in Japan (11 March 2011) occurred, the safety and economy of the current mainstream third-generation nuclear power units have been significantly improved. One of the measures is to adopt a material combination different from the existing zircaloy-clad-UO_2_ fuel system but possessing the same operating performance (burnup, power grade, abundance) as it, which aims to improve accident tolerance by transitioning from a zirconium-based fuel cladding to an excellent oxidation-resistant cladding material in the light water reactor (LWR). Although the course of the accident in LWR is controlled by the reaction of the entire (cladding-fuel) system during the accident, the accident-tolerant cladding material can significantly influence the rate of the process and consequences of the accident, which is very important to minimize the burden on the safety systems and maximize the response time. In this case, the safety margin of the nuclear power system can be improved [1]. In addition, the development of the accident-tolerant cladding is also a key issue for the high burnup operation of IV generation nuclear power system, such as the Supercritical Pressurized Water Reactor (SPWR) and the Lead Bismuth-Budding Fast Reactor (LFR). In particular, after the severe accident at Fukushima Nuclear Power Plant, nuclear reactor designers realized the importance of improving safety margins in severe accidents, and the U.S. Department of Energy started a research project on accident-tolerant fuels (fuels and fuel claddings) [2,3,4,5,6,7,8,9].

It is well understood from this extensive field of research that optimal oxidation resistance is usually achieved by forming Cr_2_O_3_, Al_2_O_3_, or SiO_2_ layers above 600 °C in air on the surface of structural materials or coatings containing Cr, Al, and Si elements [10]. Therefore, coated zirconium-based cladding, ferritic alumina-forming alloy cladding, and silicon carbide fiber-reinforced silicon carbide matrix composite cladding are considered to be the main candidates for cladding materials for accident-tolerant fuel claddings [11]. According to the research results of dry air exposed for longer than 1000 h, Cr_2_O_3_ can provide protection up to 1000 °C, Al_2_O_3_ up to 1400 °C, and SiO_2_ up to 1700 °C (the exact upper limit temperature depends on multiple factors, such as component thickness, exposure conditions, and expected life). However, under severe accident conditions, the environment inside the reactor core may consist of steam or a mixture of steam and hydrogen. In the elevated temperature environment with steam, the Cr_2_O_3_ and SiO_2_ oxide scales can form volatile hydroxides, which can reduce their service limit at high temperatures by several hundred degrees compared with the temperature limit in dry air [12]. The oxidation resistance of SiC-based materials and stainless steel supplemented with high chromium and/or aluminum was tested at temperatures ranging from 800 °C to 1200 °C in a high-pressure steam environment. The results showed that the FeCrAl alloys exhibited very low reaction kinetics up to 1200 °C; however, the Fe–Cr alloys with 15–20 wt.% Cr were corroded at a relatively high rate [13]. Thus, from the viewpoint of corrosion resistance at ultra-high temperatures (T > 1000 °C), FeCrAl-based alloys are considered to be the most desirable candidates as accident-tolerant fuel cladding in light-water nuclear reactors.

The fuel cladding is the shell to seal the nuclear fuel, serving as the most important safety barrier for nuclear power plants. Its main function is to prevent the escape of fission products, prevent the fuel from being corroded by coolant, and effectively export thermal energy. Due to the extreme service conditions of nuclear reactors, the fuel cladding is confronted with extremely high burn-up temperatures, strong radiation doses, and compatibility issues with the coolant. The fuel cladding material needs to have excellent comprehensive performance to overcome challenges such as:

(1) High thermal conductivity, low coefficient of thermal expansion;
(2) Small neutron absorption cross-section, low induced radioactivity, short radioactive half-life, and good radiation resistance;
(3) Good compatibility between the fuel and the coolant (strong corrosion resistance);
(4) High strength, good plasticity, and toughness at elevated temperatures.

Therefore, the design of chemical composition is one of the key measures for alloys to meet the service requirements of nuclear reactors.

Oxide dispersion strengthened (ODS) alloys have a strong pinning effect on the movement of dislocations and grain boundaries due to the ultra-high density dispersed nanosized oxides [14,15,16], which can significantly improve the high-temperature strength of the material [17]. High-density nanosized oxides can also promote the recombination of point defects caused by irradiation, including the recombination of vacancies and self-interstitial atoms, reducing radiation damage [18]. Due to their excellent radiation resistance and excellent high-temperature strength, ODS alloys are considered candidate materials for fast reactor fuel cladding and the first wall/cladding of a fusion reactor.

The ODS FeCrAl alloy combines the prime mechanical properties at elevated temperatures [19,20] and the radiation resistance [21,22,23,24,25] of the ODS Fe–Cr alloy with the optimized corrosion resistance of the FeCrAl alloy. Thus, it is considered a very promising candidate for solving the issue of accident-tolerance structural materials applied in nuclear energy systems, especially for the II and III generations of Lightwater Reactors (LWRs), the IV generation of Supercritical Pressurized Water Reactors (SCPWs), and Lead-Cooled Fast Reactors (LFR, using liquid lead alloy as coolant) [26,27].

**Figure 1:**
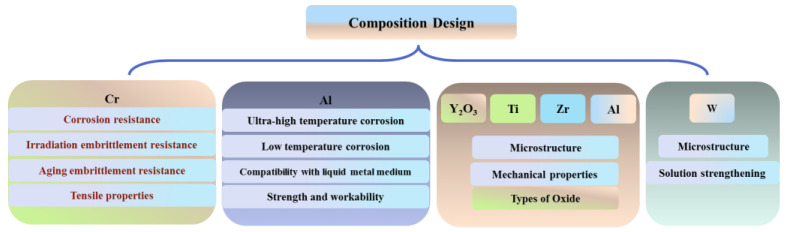
Frame diagram of composition design.

This paper reviews the historical evolution of the ODS FeCrAl alloy with respect to composition design. The roles of matrix elements (Cr/Al), oxide–forming elements (Y_2_O_3_/Ti/Zr), and solid solution strengthening element (W) in the ODS FeCrAl alloy were discussed. The effects of Cr on corrosion resistance, irradiation resistance, thermal stability, microstructure, and mechanical properties were summarized. The critical roles of Al in corrosion resistance at different conditions were reviewed in more detail. The effect of Al on the strength and workability of the ODS FeCrAl alloy were discussed in an independent section. Moreover, the upper limits of their contents were also explained on the basis of the several constraints above. The typical types of oxide, i.e., Y–Ti–O phase, Y–Al–O phase, and Y–Zr–O phase, and their formation mechanisms in alloy systems were introduced in detail in this paper. All aspects mentioned above are of reference significance for understanding the development of the ODS FeCrAl alloy for the tolerance of accidents in nuclear applications from the perspective of composition design. Figure 1 displays the frame diagram of this review, which briefly outlines the considerations of chemical composition design.

## 2. Effect of Chemical Elements on the Properties and Microstructure of ODS FeCrAl

Alloying composition or content design depends on multiple constraints in nuclear applications, such as radiation resistance, corrosion resistance, high-temperature strength requirements, processing feasibility, etc. Thus, taking measures of balancing and adjustment in the aspect of chemical composition design has always been an important part of the research and development of nuclear-level ODS FeCrAl. The effects of chemical elements such as Cr, Al, Y_2_O_3_, Ti, Zr, and W are discussed in the following content. Particularly, considering that the cladding material operates in the actual environment of SCW and LBE fission reactors with radiation and corrosive medium for the long term, it should be emphasized that Cr and Al content are determined mainly according to the results of corrosion and radiation tests [28].

### 2.1. The Influence of Cr

For Al-free ODS Fe–Cr alloys, increasing the content of Cr can improve their corrosion resistance, according to the experimental results. Pint et al. [29] compared the corrosion resistance of four ODS 13–14 at.% Cr iron base alloys in air and 10 vol.% water vapors for 10,000 h at 700–1100 °C. The results showed that the reaction rates of all ODS alloys are lower than those of stainless steel (type 347 stainless steel (18 wt.% Cr), type 310 stainless steel (25 wt.% Cr), and NF709 (Fe–20Cr–25Ni–Nb) at 700–800 °C. However, the limit temperature of alloys with a low Cr content in air is 900 °C. However, studies on ODS alloys containing 21 wt.% Cr (high content) showed that they have good oxidation resistance up to 1100 °C due to the formation of a coherent and Cr-rich oxide film [30]. Studies that refer to increasing Cr content as improving the corrosion resistance of alloys in the SCPW (783 K, 25 MPa) environment were also reported [31,32]. For example, Kimura et al. [31] concluded that in SCPW, an increase in Cr concentration to 16 wt.% significantly increases the corrosion resistance of ODS ferritic steels. Cho et al. [32] demonstrated that the higher the Cr concentration, the fewer Fe–Cr-rich corrosion products there are and the better the corrosion resistance of ODS steel when exposed to high–Cr ODS alloys in SCPW. However, the service temperature in the nuclear reactor ranges from 300 to 650 °C, and cladding material with a Cr element of 14–22 wt.% undergoes aging hardening during long thermal aging, which is called “475 °C embrittlement” [33,34,35,36]. Lee et al. [37] studied the effect of thermal aging (for 1000 h at 430–475 °C) on the microstructure and mechanical properties of ODS alloys with a high Cr content ranging from 14 to 22 wt.% by TEM, microhardness, and small punching (SP) tests. The SP-ductile to brittle transition temperature (SP-DBTT) and microhardness of ODS steel after aging increased significantly with changes in Cr content, aging time, and temperature due to the formation of the Cr–α′ rich phase.

**Figure 2:**
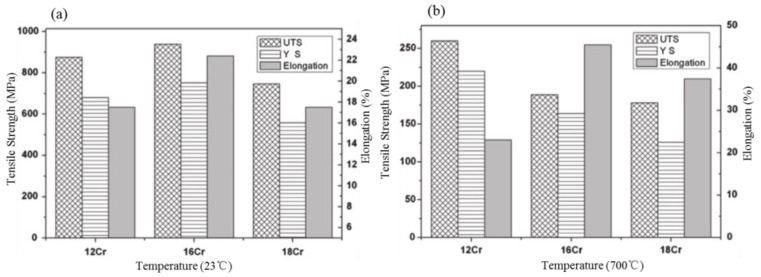
**A** comparison of tensile strength and elongation of different specimens at RT and 700 °C: (**a**) Low magnification image, (**b**) High magnification image [40].

In terms of irradiation stability, Field et al. [38] and Briggs et al. [39] reported that high Cr alloys could produce irradiation-induced harmful and embittered Cr–α′ rich phases at 300–350 °C. Therefore, commercial high-Cr ODS alloys, such as PM2000 and MA956, are not classified as nuclear-grade alloys due to aging and irradiation-induced embrittlement, although they were manifested to have satisfactory corrosion resistance. However, under moderate temperature irradiation, the reduction of Cr content hinders the formation of an embittered Cr–α′ rich phase, which can avoid damage to mechanical properties due to α′–α in the corresponding service environment [38,39]. Fortunately, the change in Cr content has no significant effect on the mechanical properties [40,41]. For example, Li et al. [40] compared three kinds of ODS alloys containing different Cr contents, i.e., Fe–12Cr–0.5Ti–1.0W (Alloy A), Fe–16Cr–0.5Ti–1.0W (Alloy B), and Fe–18Cr–0.5Ti–1.0W (Alloy C) alloys (all in wt.%). Regarding the important mechanical properties, such as tensile strength, hardness, and impact toughness, it was found that there was no linear proportional relationship between Cr content and mechanical properties on the ODS Fe–Cr alloys, as shown in Figure 2. Noh et al. [41] also concluded that Cr was the most important element in determining the matrix phase and that there was no significant influence on the tensile properties of ODS steel at 700 °C. Therefore, it is feasible to adjust the content of Cr to not only maintain the bcc matrix phase but also to avoid the emergence of a harmful brittle phase without damaging the mechanical properties.

**Figure 3:**
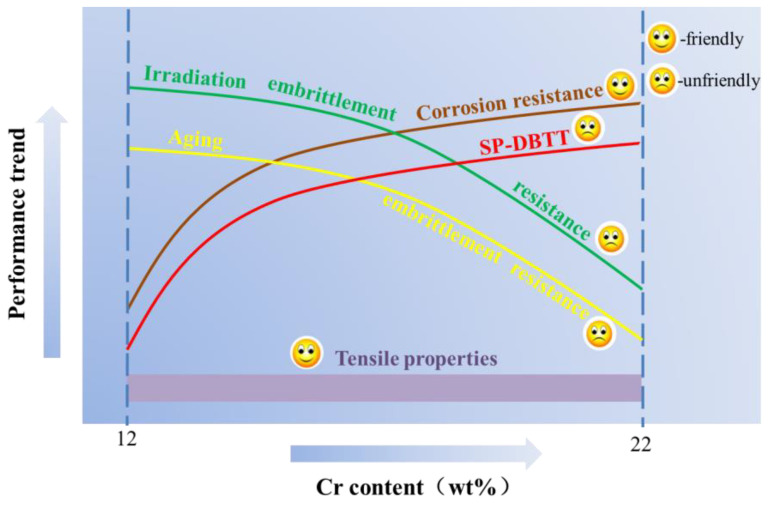
Schematic diagram of the trend of properties changing with Cr content.

As summarized above, too high a Cr content has led to many unfriendly trends in the performance of the ODS alloy, as shown in Figure 3. Therefore, reduction of Cr content is an essential measure based on the considerations of high-temperature irradiation in nuclear reactor applications [42,43,44], and an Al element is added to the ODS Fe–Cr alloy to compensate for the lack of corrosion resistance of the alloy.

### 2.2. The Influence of Al

**Figure 4:**
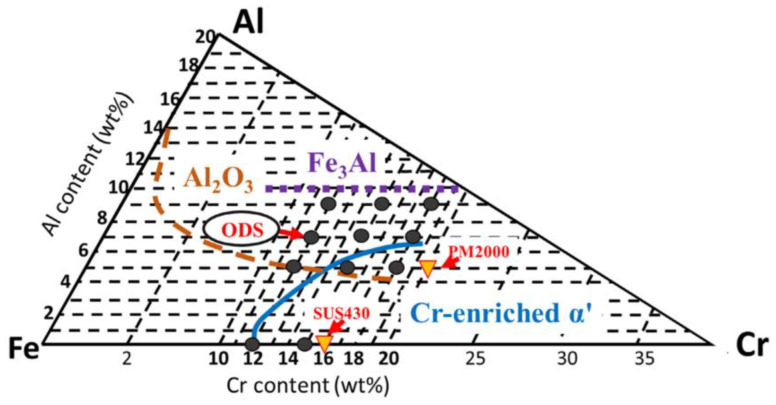
Fe–Cr–Al ternary diagram: black spots and triangles represent the compositions of the ODS steels investigated in the presented work and the commercial steels, respectively. The Al_2_O_3_ formation zone at 1100 °C is bounded by a broken line. The zones of Fe_3_Al and Cr-enriched α phases at 475 °C are bounded by a dotted line and a solid line, respectively [49].

In order to balance the weakening of corrosion resistance caused by the reduction of Cr content and considering that Al_2_O_3_ scales can withstand high temperatures up to 1400 °C to protect the underlying matrix effectively, an appropriate amount of Al addition was added to ODS Fe–Cr alloys to produce an Al–rich oxide film (Al_2_O_3_) with better performance than Cr–rich oxide film in the case of an accident. In addition, the Cr element can assist in the formation of the Al_2_O_3_ scale, that is, the third element effect. For example, Niu et al. [45] systematically studied the oxidation behavior of Fe–x Cr–3 at.% Al alloy (x = 2, 3, 5, and 10 at.%) at 1000 °C. It was found that the presence of a third element, Cr, promoted the formation of an Al_2_O_3_ oxide layer [46] in alloys with low Al concentrations. Meanwhile, the synergy effect between Cr and Al is also reflected with respect to the suppression of 475 °C embrittlement by Al addition. In terms of previous experimental observations, Kobayashi and Takasugi [47] discovered that the suppression of 475 °C embrittlement due to Al addition could be attributed to the inhibition of the nucleation of α′ phase. Regarding the simulation calculation, Li et al. [48] studied the effect of Al addition on the formation energy of the α–α′ interfaces in Fe–Cr alloy systems by utilizing the first-principles theory and suggested that Al reduced the stability and affected the formation energies of the α′ phase, resulting in suppression of phase separation in Fe–Cr alloy systems by adding Al. Theoretically, the Fe–Cr–Al ternary diagram [49] can be used as a guiding basis to prevent the generation of α′ phase with respect to chemical composition design. We can clearly understand from Figure 4 that in the Fe–Cr–Al alloy system, when the content of Cr is fixed, increasing the content of Al can effectively inhibit the generation of α′ phase.

**Figure 5:**
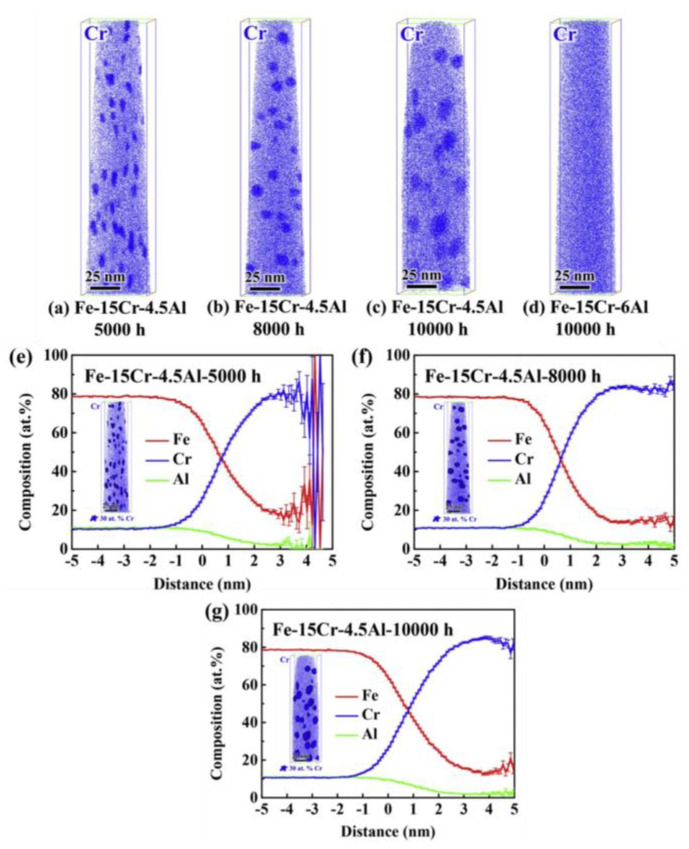
Atom probe maps of Cr for the Fe–15Cr–4.5Al alloy aged at 475 °C for (**a**) 5000 h, (**b**) 8000 h, and (**c**) 10,000 h, as well as (**d**) the Fe–15Cr–6Al alloy aged at 475 °C for 1000 h, 3D atom distributions with Cr iso–concentration surfaces at 30 at.%, and proximity histograms of the α′ phases generated by Cr iso–concentration surfaces of the Fe–15Cr–6Al alloy aged at 475 ◦C for (**e**) 5000 h, (**f**) 8000 h, and (**g**) 10,000 h [51].

Recently, some scholars have also verified the effect of the Al element on inhibition of α′ phase [50,51]. For example, Yang et al. [51] characterized α′ phase evolution by the atom probe, which evolved an entire process of nucleation, growth, and coarsening of α′ phase during aging at 475 °C. The results showed that in the case of 15 wt.% Cr, the size of α′ phase in the model alloy containing 4.5 wt.% Al grows gradually with the aging time up to 10,000 h, while α′ phase in the model alloy containing 6 wt.% Al is effectively suppressed, as shown in Figure 5. These experimental observations are in good agreement with the theoretical prediction displayed in Figure 4.

The combined addition of Cr and Al is the key to the corrosion resistance design of alloys, and the Cr and Al content are determined by the corrosion test results in SCW and LBE. This section focuses on the evaluations for the corrosion resistance of the ODS FeCrAl alloy at ultra-high temperatures under simulated accident conditions (Section 2.2.1), normal operating temperatures (Section 2.2.2), and in lead–bismuth eutectic alloy (LBE) (Section 2.2.3) to emphasize the critical role of Al in corrosion resistance. In addition, the influence of Al content on strength, workability, and aging brittleness was summarized (Section 2.2.4). It should be noted that as for research on the corrosion or oxidation resistance of the ODS FeCrAl alloy, some researchers have considered the synergistic effect between Cr and Al elements, while some other studies only evaluated the influence of Al content on corrosion resistance.

#### 2.2.1. Ultra-High Temperature Corrosion (T > 1000 °C)

**Figure 6:**
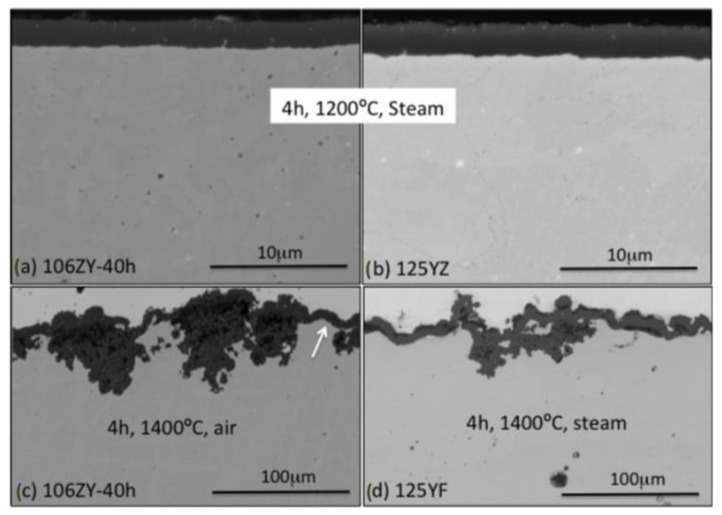
BSE-SEM micrographs of the oxide scale formed (**a**) after 4 h at 1200 °C in steam, alloy 106ZY 40 h; (**b**) after 4 h at 1200 °C in steam, alloy 125YZ; (**c**) after 4 h at 1400 °C in air, alloy 106ZY 40 h; (**d**) after 4 h at 1400 °C in steam, alloy 125YF. The scale bars for (**c**,**d**) are ten times larger than the scale bars for (**a**,**b**). The white arrow highlights an area where the scale is locally protective [60].

As for the high-temperature corrosion studies of the ODS FeCrAl alloy, scientists are most especially concerned about the corrosion behavior of the alloys under simulated accident conditions, i.e., loss-of-coolant accidents (LOCA). To prove the important role of the Al element, this section reviews the corrosion behavior of the ODS FeCrAl alloy at ultra-high temperatures up to 1621 °C [52,53]. Liu et al. [54] carried out oxidation tests on six ODS ferritic alloys and SUS430 at 1050 °C for 200 h in air. The results showed that the oxidation resistance of the 14Cr ODS FeCrAl alloy containing Al elements was better than that of SUS430 (16.8Cr). With an increase in Al concentration, the oxidation resistance of the 16Cr ODS alloy was significantly improved from 0 to 2 and 4 wt.%, and the mass gain decreased from 8.6 to 7.6 and 4 g/m^2^. The thickness of the Al_2_O_3_ oxide layers of the 16Cr–4Al ODS alloy can only be 3.5 μm. While ODS alloys containing 11 wt.% Cr and 2 wt.% Al cannot form a dense alumina layer. The concentrations of Al and Cr should be greater than 2 and 14 wt.%, respectively, to form a dense Al_2_O_3_ layer and enhance adherence between the Al_2_O_3_ layer and the matrix. The author [55] studied systematically the effect of Al content on the oxidation behavior of Y_2_Ti_2_O_7_ dispersed Fe–14Cr ferritic steel in air at 1100 °C for 200 h. It was concluded that in 14CrODS steel containing 14 wt.% Cr, a solid and continuous Al_2_O_3_ film is formed, and the content of Al is not less than 4.5 wt.%. Furthermore, previous studies also showed that a reduced Cr content and enhanced Al content provide the basis for the formation of a stable and protective Al oxide layer [56,57]. In a Fe–Cr–Al alloy with 1015 wt% Cr, an Al content as low as 3 wt.% is sufficient to form an Al oxide layer. The Oak Ridge National Laboratory (ORNL) in the United States developed the first generation 12Cr ODS FeCrAl alloy (low Cr), and the evaluating experiment started with oxidation performance in simulated accident status at 1200 °C for 4 h in 1 bar dry air or steam. All evaluated components, including 12Cr–5Al, formed a protective film in steam at 1200 °C, while the ODS Fe–14Cr alloy did not form a protective oxide film under the same experimental conditions, and metal consumption was 50% after 8 h in this environment [58,59]. Subsequently, in order to improve the ductility of the alloy, the second-generation Fe–(10–12)–Cr–6Al ODS alloys with a low O content and 0.15–0.5 wt.% Zr were derived from the first-generation low Cr12–15Cr ODS FeCrAl alloy in ORNL. The experimental results of the isothermal oxidation test up to 1400 °C in air or steam showed that this series of alloys have excellent corrosion resistance above 1200 °C under simulated accident conditions, as shown in Figure 6 [60].

Qiao et al. [61] calculated the corresponding Gibbs free energy of different oxide layers at 1200 °C by Fact Sage. The priority order of each element that forms oxides in the Fe–Cr–Al alloy can be predicted as Al_2_O_3_ > Cr_2_O_3_ > FeCr_2_O_4_ > Fe_2_O_3_ > FeO > Fe_3_O_4_, thus Al will take precedence over Cr and Fe oxidation, which is consistent with previous experimental results.

**Figure 7:**
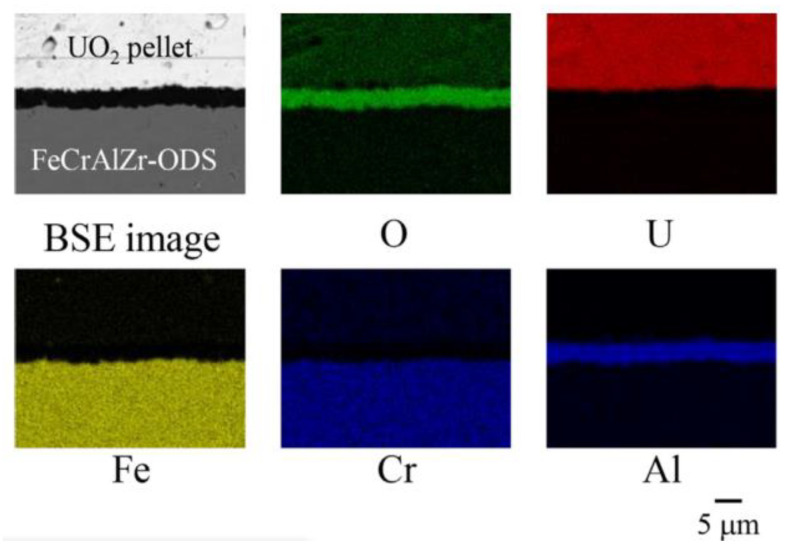
Elemental mappings on the cross-section of the reaction couple of FeCrAlZr–ODS–UO_2_ at 1723 K for 25 h [53].

Regarding oxidation resistance studies under simulated accident conditions, the research team of Hokkaido University in Japan evaluated systematically the oxidation behavior of a series of the ODS 15Cr–7Al alloys in air and steam at 1200–1500 °C, simulating the LWR severe accident together with analyses using the thermochemical multiphase computer software FactSage (https://www.factsage.com/, 11 September 2023). Additionally, they found that the alumina scale formed on the ODS FeCrAl alloy with an appropriate composition can maintain good adhesions up to 1400 °C, which indicated that the ODS FeCrAl alloy is capable of accident-tolerant fuel (ATF) clad material [52]. Further, Sakamoto, Miura, Ukai, et al. [53] conducted reaction tests between ODS FeCrAl and UO_2_ in an inert gas atmosphere at 1723 K for 25 h. The Al_2_O_3_ scale with a thickness of 3.6 µm was formed, and no detectable inter-diffusion across the oxide scale was found, as shown in Figure 7. More research [62] showed that the growth rate of the Al_2_O_3_ reaction layer of the FeCrAlZr-ODS with UO_2_ was evaluated to be 10^−8^–10^−7^ m^2^ s^−1^, while the rate of the reaction layer between zircaloy and UO_2_ was extremely large at the same temperature (10^−2^–10^−1^ m^2^ s^−1^), indicating good compatibility.

Table 1 summarizes the recent experimental data on the ODS FeCrAl alloys (SUS430 as a comparison) after long-term and short-term oxidation tests at ultra-high temperatures (T > 1000 °C). Data show that ODS FeCrAl alloys with optimized composition (3.5 μm of oxide layer thickness) exhibit better oxidation resistance than SUS430 without Al (20 μm of oxide layer thickness) after a long-term oxidation test of 200 h at high temperatures below 1200 °C [54,55]. Furthermore, after exposure to a short–term oxidation test at 1200 °C, the ODS FeCrAl alloy can produce protective and stable α-Al_2_O_3_ with a thickness of approximately 2 μm, which is negligible for the fuel cladding tube with a thickness of 500 μm [58,59,60]. In the reaction experiment [52,53], where the temperature is close to the melting point (mp), Al_2_O_3_ formed on the ODS FeCrAlZr alloy can still keep the alloy isolated from the UO_2_ pellet, which greatly reduces the reaction rate between the fuel cladding and fuel pellet. As a result, the damage to the fuel cladding tube’s mechanical properties caused by oxidation can be reduced to the lowest level due to the protection provided by Al_2_O_3_.

#### 2.2.2. Low Temperature Corrosion in a Light Water Nuclear Reactor (T < 1000 °C)

**Figure 8:**
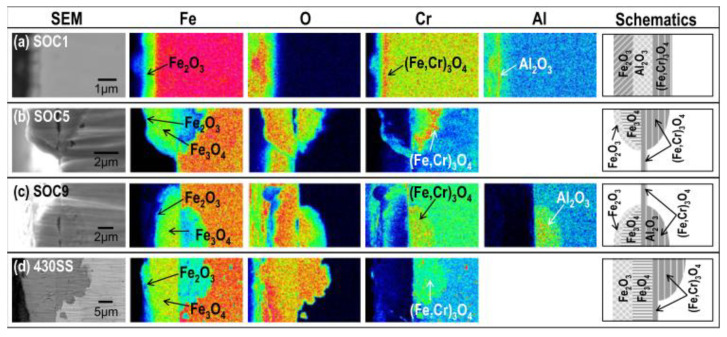
SEM images showing the cross-sectional morphologies of (**a**) SOC-1 (Fe–16.11Cr–3.44Al–0.09Ti–0.34Y_2_O_3_), (**b**) SOC-5 (Fe–15.95Cr–0.09Ti–0.34Y_2_O_3_), (**c**) SOC-9 (Fe–15.42Cr–1.85W–3.8Al–0.10Ti–0.36Y_2_O_3_), and (**d**) type 430 SS (Fe–16Cr) and FE-EPMA analyses showing the corresponding composition distributions after exposure at 500 °C [64].

The normal operating temperature in II and III Generation light water nuclear reactors ranges from 290 to 320 °C, while in the IV generation of supercritical pressurized water reactor (SCW), the operating temperature increases from 290 to 600 °C. Therefore, the corrosion behavior of the ODS FeCrAl alloy in the lower temperature range of 200–700 °C over the long term has also been widely studied. Isselin et al. [63] compared the corrosion behavior of 16Cr–4Al ODS and 16 Cr ODS steel in a SCW environment (pure water, 25 MPa, 8 ppm) at 550 °C and found that the addition of 4 wt.% Al can effectively improve corrosion resistance. A dense protective layer of Al_2_O_3_ is formed on the surface of 16Cr–4Al ODS, while (Cr, Fe)_2_O_3_ is formed on the surface of 16 Cr ODS steel. Lee et al. [64] studied systematically the corrosion behavior of ODS steel doped with various alloy elements, such as Cr, Al, W, Ce, Hf, and Zr, in SCW (25 MPa and 8 ppm dissolved oxygen) at different temperatures of 400, 500, and 600 °C. The research revealed that the addition and content of different chemical elements lead to different corrosion products resulting from different corrosion mechanisms, as shown in Figure 8, and Al addition led to a dense protective layer of Al_2_O_3_ among complex corrosion products. It was also found that the thickness of the oxide layer is also closely related to the exposure temperature and time, which is due to the different diffusion coefficients of the O atoms at different temperatures. The resistance to corrosion at low temperatures also depends on the content of the Al element. For example, exposed to SCW at 600 °C for one year, the oxide thickness of SOC-1 (with a higher Al of 3.5 wt.%) was only 5 μm. It showed that 4 wt.% Al addition can effectively improve the corrosion resistance of 16Cr–ODS, and the appropriate composition ratio of Cr and Al is in the range of (14–16) Cr and (3.5–4.5) Al for SCW application. Briefly, in SCW, it can be seen that Al addition and content strongly control the corrosion rate, corrosion products, and corrosion mechanism of the alloy.

**Figure 9:**
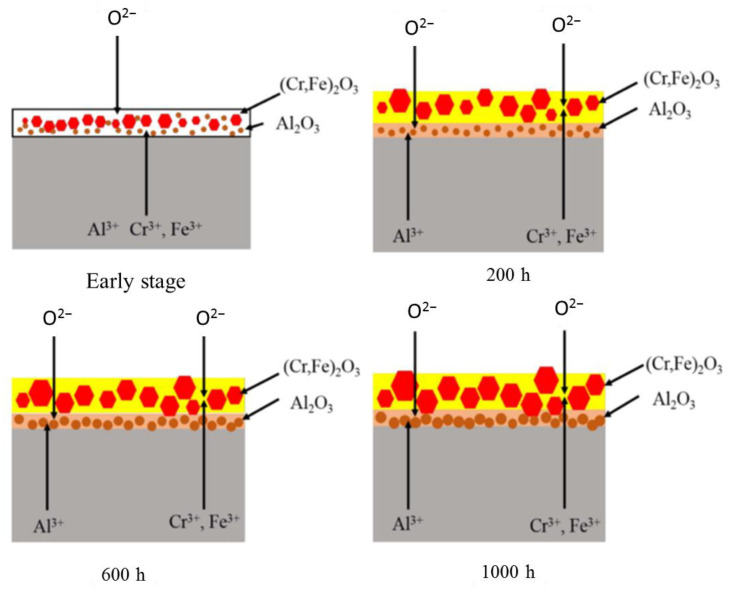
Schematic representation of the microstructural evolution of the oxide layer at an early stage in SCW and after exposure for 200 h, 600 h, and 1000 h, respectively [65].

Ren et al. [65] systematically investigated the effect of corrosion time (200, 400, 600, 800, and 1000 h) on the corrosion behavior of the 16Cr–3Al ODS alloy in SCW at 600 °C. It is concluded that with the extension of corrosion time, the thickness of the oxide layer and weight gain agree with the parabolic law in the case of the types of corrosion products without changing. On the basis of these results, the corrosion mechanism under this experimental condition was discussed in detail, as schematically shown in Figure 9. Simply put, the lower standard Gibbs free energy [66] of Al_2_O_3_ compared with Cr_2_O_3_ and Fe_2_O_3_, the difference in concentration of the elements in the alloy, the chemical gradient, and the difference in the ion diffusion coefficient make it form a double-layer oxide structure with Al_2_O_3_ as the inner oxide layer and (Cr, Fe)_2_O_3_ as the outer oxide layer. Finally, due to the densification of Al_2_O_3_ and the low diffusion coefficient [67] of Fe and Cr in Al_2_O_3_, the outward diffusion of Cr^3+^ and Fe^3+^ was obviously limited, and the growth of the outer oxide layer slowed. Under the circumstances, the process of oxidation became stable as a result of the protection of the compact internal Al_2_O_3_ layer, resulting in the prevention of corrosion damage.

**Figure 10:**
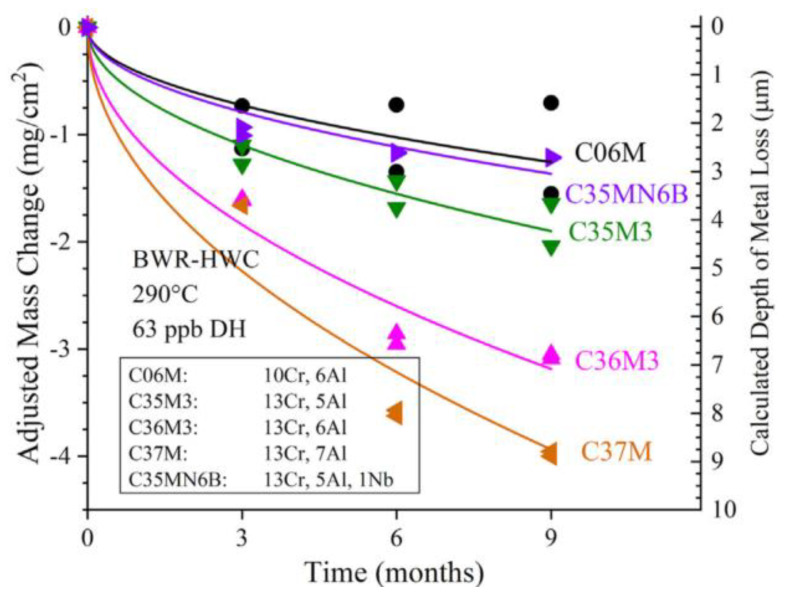
Adjusted mass change of 2nd generation FeCrAl alloys exposed to BWR-HWC (290 °C, 62 wppb DH) with fitted curves [69].

In ORNL of the United States, Terrani et al. [68,69] conducted the corrosion test for several low Cr FeCrAl alloys in water with three different chemical composition environments (found in the main cooling cycle conditions of PWR and BWR) for up to one year in a temperature range of 290–330 °C. The maximum thickness loss of LWR corrosion for one year without irradiation is 2 μm, which can be ignored for cladding with 300–500 μm thickness in actual application. Recently, a corrosion resistance test for a second generation of several FeCrAl alloys was conducted in simulated BWR-HWC and BWR-NWC under normal operating conditions [69]. The thickness of the oxide layer on Fe–10Cr–6Al increased most slowly after exposure to the corrosive environment of BWR-HWC (as shown in Figure 10) and exhibited satisfactory corrosion resistance in the corrosive environment of BWR-NWC.

Table 2 summarizes the data from the non-irradiation oxidation experiment in SCW at normal operating temperature (T < 1000 °C). According to previous research, the corrosion products of ODS FeCrAl alloys are different, which may be due to differences in components, preparation conditions, corrosion time and temperature, and types of corrosion medium, but their corrosion resistance exhibits a significant advantage when compared with commercial 430 SS according to the thickness of the oxide layer. Additionally, with an increase in Al content, the oxidation resistance was significantly improved.

#### 2.2.3. Compatibility with Liquid Metal Medium

**Figure 11:**
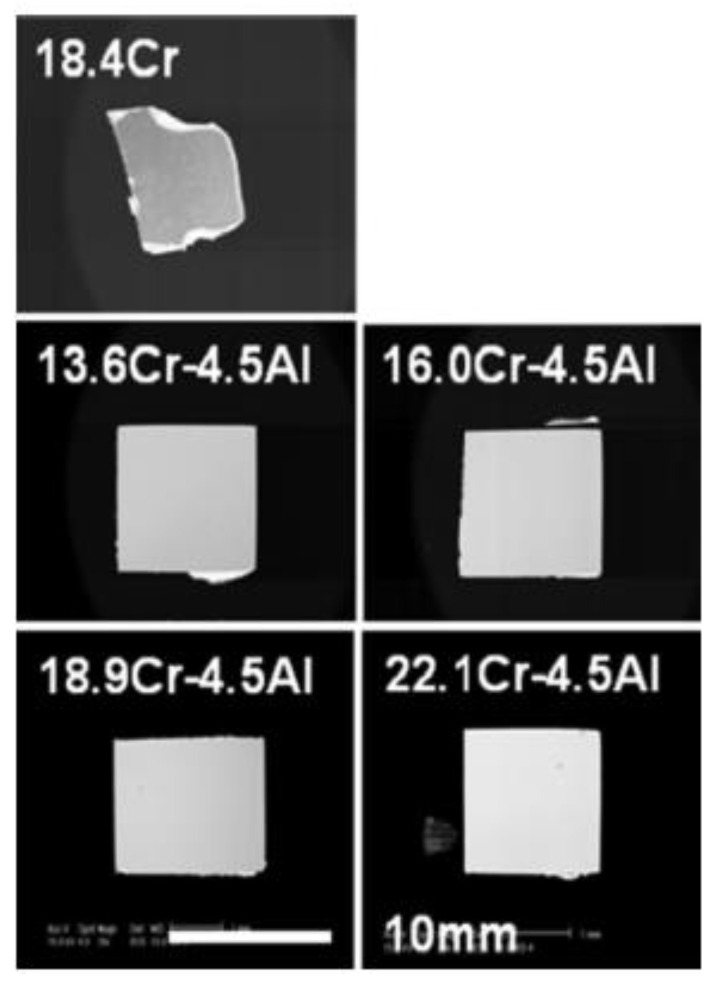
The appearance of ODS steel specimens after corrosion tests in LBE solved with 10^−6^ wt.% for 1 × 10^4^ h at 923 K [28].

Al plays a critical role in the corrosion resistance of candidate alloys in liquid metal. There exists a contradictory fact that Ni/Fe/Cr elements are very important in austenitic stainless/ferrite steel, but lead–bismuth eutectics (LBE) have high solubility for the above three elements. So, Ni superalloy and Fe–based austenitic stainless steel are difficult to utilize as structural materials for the LBE cooling system, especially above 773 K. Above 873 K, the solubility/corrosion of Fe and Cr in LBE increase remarkably. In order to keep the material from dissolving in LBE, the Al_2_O_3_ layers that form on the surface of the Al-containing steel are considered to inhibit the dissolution. Figure 11 shows ODS samples under certain LBE conditions (10^−6^ wt.% O_2_ in solution for 10^4^ h at 923 K). The 19Cr–ODS steel without Al dissolves significantly in LBE. However, the ODS samples with 4 wt.% Al retain almost completely their shape, indicating high corrosion resistance. It should be noted that with the same Al content, the change in Cr content (13–19 wt.%) does not have a remarkable impact on the corrosion resistance of the alloys in LBE [28].

Hosemann et al. [70] compared the corrosion resistance of five different ODS experimental and commercial alloys, including Fe–Cr ferritic/martensitic steels (12YWT, 14YWT, and MA957) and FeCrAl alloys (PM2000 and MA956), under the conditions of liquid lead–bismuth eutectic at 535 °C for 200 h and 600 h. The results showed that PM2000 with an Al content greater than 5.5 wt.% exhibits good corrosion/oxidation resistance, which is closely related to grain size. Takaya et al. [71] systematically studied the corrosion resistance of ODS steel with an Al content of 0–3.5 wt.% and a Cr content of 13.7–17.3 wt.% and a 12Cr steel. The results showed that when the Al content is 3.5 wt.% and the Cr content is 13.7–17.3 wt.%, a protective Al oxide layer can be formed on the surface of ODS steel, while increasing the Cr concentration alone cannot improve the corrosion resistance. These research conclusions show that Al plays a decisive role in the corrosion resistance of the alloy in the lead–bismuth eutectic environment.

**Figure 12:**
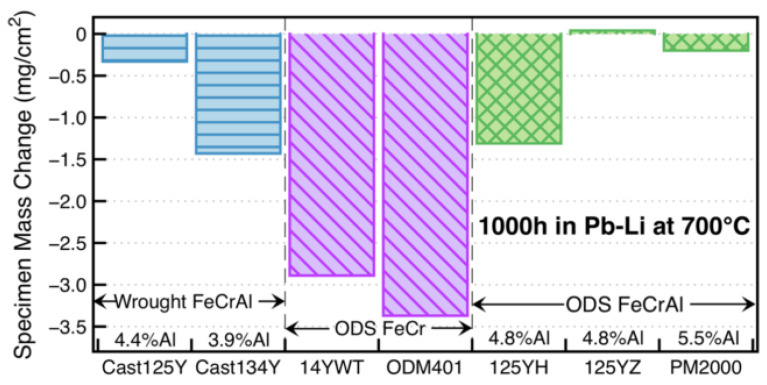
Specimen mass change for alloy specimens exposed for 1000 h at 700 °C in static PbLi [58].

**Figure 13:**
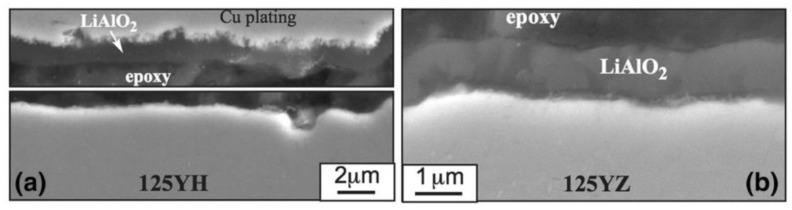
SEM backscattered electron images of polished cross-sections after 1000 h in Pb–Li at 700 °C of (**a**) 125YH and (**b**) 125YZ. In (**a**), the oxide delaminated during specimen preparation [58].

Regarding Pb-Li compatibility in the fusion reactor, Unocic et al. [72] exposed model FeCrAlY alloys with 10–20 wt.% Cr and 3–5 wt.% Al for 1000 h at 700 °C. The results showed that the effect of Cr content on mass change was not obvious; however, mass losses happened to alloys with <5 wt.% Al in these experiments, indicating that Al plays a more important role than Cr in the Pb–Li coolant. The corrosion experiment for the first generation of low Cr ODS FeCrAl (based on Fe–12Cr–5Al) developed by the Oak Ridge National Laboratory [58,59,73] has been completed at 700 °C. It showed that the Pb–Li compatibility of the ODS FeCrAl alloys is better than that of the wrought FeCrAl and ODS Fe–Cr alloys (Figure 12). A thin (~1 μm) LiAlO_2_ reaction product was detected in all cases (Figure 13), indicating that the corrosion resistance of low Cr ODS FeCrAl meets the service requirements of the liquid lead–bismuth eutectic reactor.

The historical evolution of the development of the ODS FeCrAl alloy summarized above shows that the low Cr and high Al ODS FeCrAl alloy with a certain ratio has excellent application prospects in the light water-cooling medium (fission reactor) or metal cooling medium (fission/fusion reactor) from the perspective of corrosion resistance.

#### 2.2.4. Effect of Al on Strength and Workability

The addition of Al has a significant effect on the dispersed oxides and mechanical properties of ODS steel [74]. For example, Kasada et al. [75] studied the microstructure and tensile properties of oxidation dispersion strengthened (ODS) ferritic steels K1 (F–19Cr–0.3W–0.3Ti–0.3Y_2_O_3_) and K4 (Fe–19Cr–4Al–2W–0.3Ti–0.3Y_2_O_3_) before and after deformation. The nano-oxides in K1 were cubic chlore Y_2_Ti_2_O_7_, and the nano-oxides in K4 were mainly perovskite YAlO_3_ with a larger size than Y_2_Ti_2_O_7_, which indicated that the addition of Al changed the type and average size of the ODS particles and resulted in a much higher strength of K1 (Al-free) than that of K4 (Al-containing). In similar studies, Gong et al. [76] prepared two ODS ferritic alloys with compositions (wt.%) of Fe–14Cr–4.5Al–1W–0.35Y_2_O_3_ (14Cr–Al ODS) and Fe–14Cr–0.5Ti–1W–0.35Y_2_O_3_ (14Cr–Ti ODS) by mechanical alloying and hot isostatic pressing. Tensile tests showed that 14Cr–Ti ODS steel has higher strength but weaker ductility, while 14Cr–Al ODS alloy has lower strength but better ductility. In addition, 14Cr–Al ODS alloy shows a higher impact energy than that of 14Cr–Ti ODS alloy. A similar behavior in mechanical properties was reported in references [28,77,78]. The solution to the problem of reduction in mechanical properties is to add the Zr element to the ODS FeCrAl alloys, which will be detailed in Section 3.4.

Except for a reduction in tensile strength due to the addition of Al, the higher Al content increases the difficulty of welding and manufacturing [79,80,81] and increases the brittleness of the alloy simultaneously [82,83]. Moreover, investigation [84] revealed that ordered Fe_3_Al intermetallic phase precipitating during aging increases the strength and decreases the ductility of aged ODS FeCrAl ferritic alloys when the amount of Al exceeds 10 wt.%. Thus, the upper limit of Al that can be safely added to ODS FeCrAl ferritic alloys for accident-tolerant fuel cladding is on the order of 7~8 wt.%. Thus, it can be concluded that these experimental data provide the basis for the maximum amount of Al addition in the processing or service of the alloy. In addition, it should be noted that when ODS FeCrAl alloys are exposed to oxygen and air at 1000–1300 °C, Al depletion will occur in the matrix. Therefore, the change in the Al concentration in the matrix can determine the service life of these alloys; that is, when the aluminum content is less than a critical value, the oxidation resistance of the alloys is limited. The protective layer of alumina can no longer heal, so the matrix oxide products are rapidly formed, which results in rapid consumption of materials. This phenomenon is called “separation oxidation”, which corresponds to the end of material life. When scale peeling occurs, especially in the thermal cycle, this phenomenon will accelerate [85]. This phenomenon of “separation oxidation” should be avoided during thermal mechanical processing because it may make the material unable to meet the corrosion resistance requirements and fail in normal service or accident situations.

## 3. Influence of Elements on Microstructure and Properties

Different chemical element additions produce different effects on the microstructures and mechanical properties of the alloy. The types and size distribution of complex oxide particles, i.e., Y–Ti–O, Y–Al–O, and Y–Zr–O phases, in ODS FeCrAl alloys are significantly influenced by different oxide-forming elements due to different free energy and element content. The influence trends of elements such as Y_2_O_3_, Ti, Al, and Zr on microstructure and performance, as well as their internal formation mechanisms, will be introduced in this section. In addition, two special precipitates, namely co-precipitation and core–shell structure, are summarized in detail in this section, and the formation mechanisms of specific precipitates are briefly reviewed.

### 3.1. Y_2_O_3_

Dispersed Y_2_O_3_ was first utilized in ODS alloys due to its thermodynamically more stable free energy of formation than the sulfides, nitrides, and carbides phases [86,87]. Additionally, Y_2_O_3_ also acts as a strong obstacle for mobile dislocations and serves as a sink for radiation defects at the oxide-matrix interfaces to improve high-temperature mechanical strength and ductility by controlling the structure of grain boundaries [86,88,89,90]. The advantage of ODS alloys is that the dispersed oxide is uniformly dispersed and shows limited solubility or good coarsening resistance, which gives the alloy a higher strength at an elevated temperature. The ability of the dispersed phases to produce and retain coarse-grained alloy structures leads to increased creep resistance at elevated temperatures [91] and improves the mechanical strength of the alloy, such as hardness [92], tensile property [93], and impact properties [94], but also improves the oxidation resistance through the reactive element effect (REE) [95,96,97] For example, Cueff et al. [96] studied the oxidation behavior of FeCrAl alloys in air at 1173 K. The alloys differ in whether yttrium is implanted. The kinetic results showed that in the early stage of oxidation, only yttrium implantation can significantly reduce the growth rate of the oxide layer. For a longer oxidation time, the active elements significantly affect the oxidation rate and the composition of the oxide film, regardless of the way they are introduced into the alloy. Yttrium suppressed the formation of transition alumina, promoted the growth of α-Al_2_O_3_, and resulted in a protective oxide layer earlier. UI-Hamid [97] studied the cyclic oxidation behavior of two ODS FeCrAl alloys, MA956, containing 0.17 and 0.7 Y_2_O_3_ by mass percentage. The alloy was exposed to air at 1200 °C for 100 h on a 24 h cycle. Both alloys form a highly adherent continuous α-Al_2_O_3_ layer, whose morphology indicates inward scale growth. At the same time, Y_2_O_3_ produces micropores in a 0.7 wt.% alloy, thus forming a relatively high thickness and high oxidation rate, which indicates that Y_2_O_3_ should be kept below an appropriate content in terms of oxidation resistance. However, to adapt to the development of ODS FeCrAl alloys in nuclear reactors, the Y_2_O_3_ usually added is decomposed in the mechanical alloying process and then reacts with other oxide-forming elements, such as Ti, Zr, etc., in the subsequent thermal curing process (HIP, HE, SPS, etc.), to form finer oxides in the matrix rather than coarser Y_2_O_3_ particles. Details will be discussed in the following sections.

### 3.2. Ti

**Figure 14:**
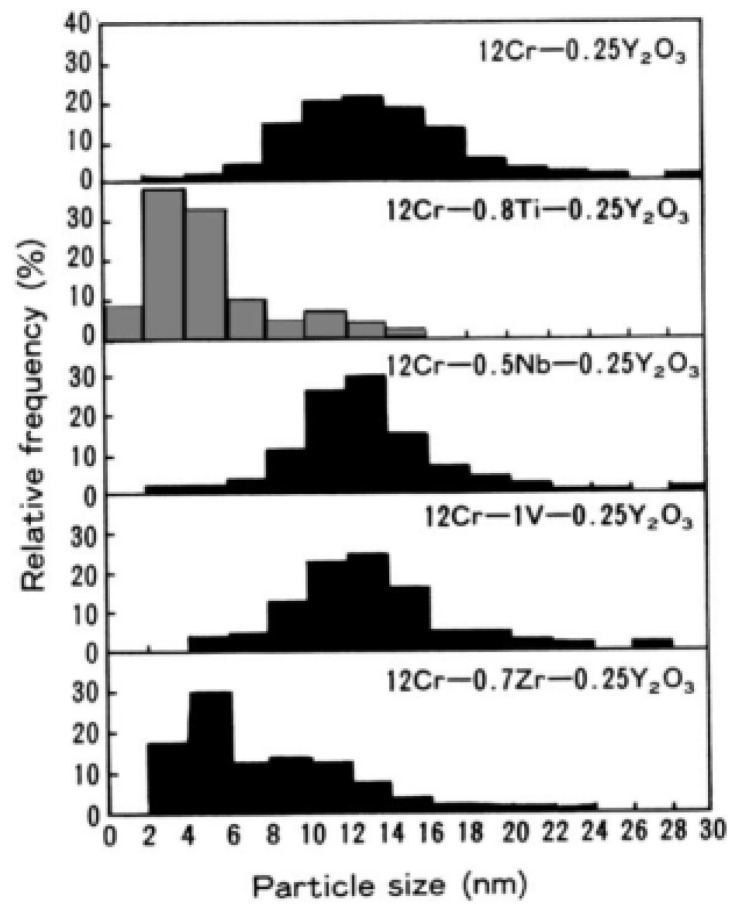
Size distribution of oxide particles determined by TEM in 12Cr–ODS ferritic steels in various element additions: Ti, Nb, V, and Zr [101].

**Figure 15:**
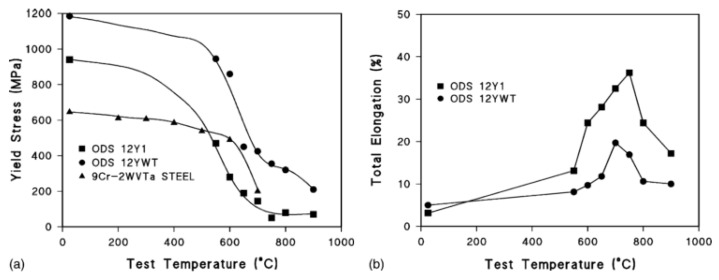
(**a**) The 0.2% yield stress of the 12Y1 and 12YWT ODS steels and the reduced-activation 9Cr–2WVTa steel, and (**b**) the total elongation of the 12Y1 and 12YWT [110].

**Figure 16:**
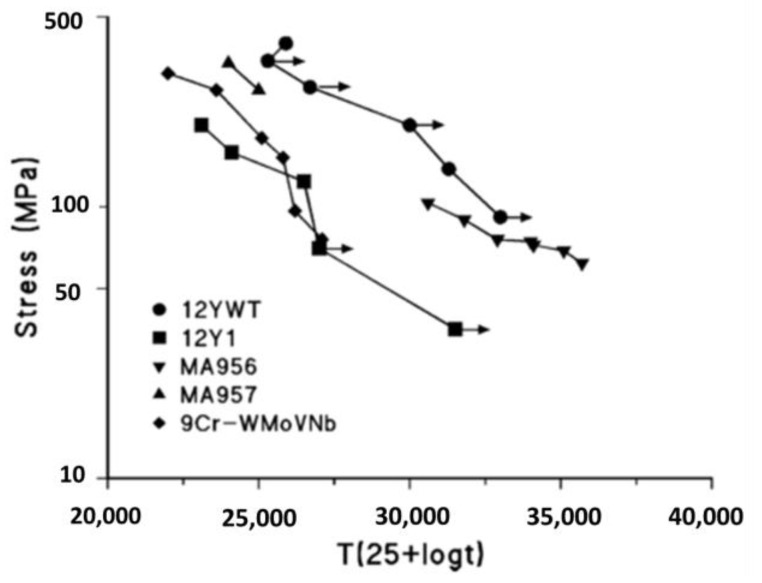
Larson–Miller diagram for the creep–rupture strength of four ODS steels and a conventional ferritic/martensitic steel. The arrows indicate that the test is still in progress or that it was discontinued prior to rupture [110].

Regarding the ODS Fe–Cr alloy without Al, Ukai et al. [98,99] recognized the most effective and important role of Ti in the refinement of dispersed particles by systematically analyzing the effect of the addition of elements, such as Ti, Nb, V, and Zr, on the Al-free 12Cr ODS ferrite alloys, as shown in Figure 14. The main function of the Ti element is to generate finer Y–Ti–O particles through the reaction of TiO_2_ and Y_2_O_3_, which results in a decrease in oxide size and an increase in number density [100]. Detailed research showed that Y_2_Ti_2_O_7_ oxide (~2 nm) is much smaller than Y_2_O_3_ (>10 nm) [101,102,103]. Additionally, the main crystal structures of dispersed particles are Y_2_Ti_2_O_7_ and Y_2_TiO_5_ (2–30 nm) [104,105,106,107,108,109]. By adding Ti to Al-free 12Cr–ODS alloys, tensile and creep properties, as shown in Figure 15 and Figure 16 [101,110], were significantly improved compared to Al-free 12Cr–ODS without Ti due to the reduced size of oxide particles. However, in this case, the effect of Zr on the reduction of oxide size is weaker than that of Ti, which is accompanied by a small increase in creep rupture strength [101]. As a result, Zr has not been popularized in Al-free Fe–Cr ODS alloys.

In science, then, Al-free Fe–Cr ODS alloys containing Ti have been extensively studied, the results of which showed that Y–Ti–O phases exist universally. For example, Sakasegawa et al. [111] found a non-stoichiometric pyrochlore Y_2_Ti_2_O_7_ with a cubic lattice in the MA957 by high-resolution TEM (HRTEM). The Y_2_Ti_2_O_7_ oxides coherent with the matrix were also detected in Fe–14Cr–1W–0.3Ti–0.3Y_2_O_3_ [112]. The findings above are consistent with most of the complex oxides detected by Yamashita et al. [102] in a Fe–12Cr–1.9W–0.3Ti–0.24Y_2_O_3_ alloy and Kishimoto et al. [113] in Fe–16Cr–0.1Ti–0.35Y_2_O_3_. In addition, there are also phases like Y_2_TiO_5_ with the structure of an orthorhombic lattice or a thogonal lattice [102].

The formation of complex oxides is based on the large density of vacancies formed in the alloyed powder during mechanical alloying. Zhang et al. [114] investigated the variation in the density of vacancies during mechanical alloying (MA) and proposed a model for the evolution of vacancies. Results of the simulation showed that, to some extent, the density of vacancies increases with longer collision times, lower milling temperatures, and higher collision frequencies. The results of positron annihilation life-time studies [115,116,117] proved that four to six vacancies were contained in the existence of vacancy clusters in the mechanically alloyed (MA) and extruded ODS ferritic alloys. A vacancy mechanism underlying the unusually high O solubility and nucleation of stable O-enriched nanoclusters in defect-containing Fe was widely accepted from the results of first-principles studies [118,119]. Oxygen, as an interstitial, shows an excellently high affinity with vacancies; the interaction between O and Y in Fe had to be mediated by vacancies; without vacancies, the interaction between O and Y in Fe was found to be repulsive. Thus, the O-vacancy mechanism enables the nucleation of O-enriched nanoclusters that attract solutes with high O affinities (Ti and Y) and strengthen Fe-based alloys [118]. By composition-sensitive APT and size-sensitive SANS experiments, Zhang et al. [120] reported the existence of a large number of vacancies in NCs as a constitutive element and concluded that the nucleation of nanoclusters starts from the O-enriched solute clustering with vacancy mediation. Regarding the forming priority of O–Y–Ti clusters compared with O–Y clusters, Vallinayagam et al. [121] investigated six different structural models for atomic clusters in bcc Fe by density functional theory (DFT) calculations, which showed that the driving force for the growth of O–Y clusters should be greater than that of O–Y–Ti clusters. This could be correlated with the experimental observation that the presence of Ti leads to a reduction in the size of the oxide clusters in nanostructured ferritic alloys and a higher dispersion. A model framework for the thermodynamics and kinetics of Y–Ti oxide nucleation, growth, and coarsening was developed by Barnard et al. [122]. Based upon the available thermodynamic and kinetic data as well as key density functional theory calculations, the model shows that the nucleation and growth of nano-oxide are highly driven and that pipe diffusion is the dominant mode of their coarsening, consistent with the previous conclusion from experimental high-temperature data. This analysis also provides insights into the effect of O and Ti on nano-oxide sizes and the optimization of the alloy microstructure. In similar work, Boulnat et al. [123] modeled the precipitation behavior of nanosized binary Y_2_O_3_ and complex Y_2_Ti_2_O_7_ oxides in ODS ferritic alloys by a nucleation, growth, and coarsening thermodynamic approach. The precipitation state was characterized at the nanoscale by transmission electron microscopy (TEM), small-angle neutron scattering (SANS), and atom-probe tomography (APT). Both simulation and experimental analysis showed the precipitation mechanisms of two steps: the first step is rapid nucleation of nanoclusters during the heating stage; the second step is limited growth and coarsening of Y_2_O_3_ and Y_2_Ti_2_O_7_ during the soaking time and further annealing at elevated temperature (1100 °C).

**Figure 17:**
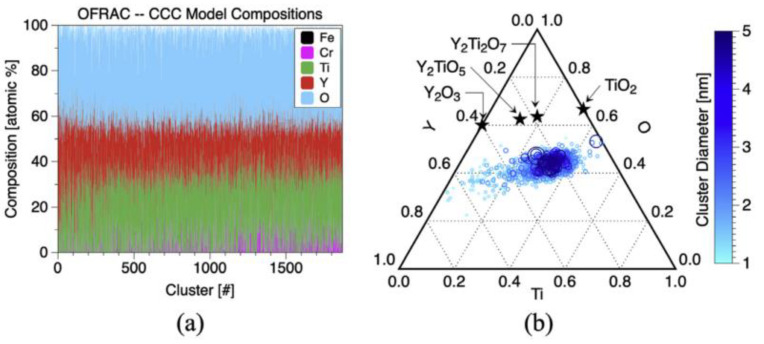
Visualization of (**a**) individual cluster compositions for all 1871 nanoprecipitates analyzed in the APT control volumes in this work and (**b**) the ternary Y-Ti-O composition diagram as a function of cluster size [126].

For a suitable amount of addition, research showed that excessive Ti leads to coarsening of oxide particles in the alloy, as shown in Figure 6 of Ref. [124], or produces potential embrittlement due to large TiO_2_ particles [125], which will damage the mechanical properties. The ternary Y–Ti–O composition diagram [126] well explains the influence of the Ti content on the type of Y–Ti–O phase. As shown in Figure 17b, with increasing Ti content, the type of Y–Ti–O phase changes from Y_2_TiO_5_, Y_2_Ti_2_O_7_, to TiO_2_. Thus, in this alloy system, there should be a reasonable optimal value below 0.5 wt.%.

### 3.3. Al

In Zr-free ODS FeCrAl, when adding an Al content (≥5 wt.%) to the ODS alloy containing the Ti element (~0.3 wt.%), the strengthening effect reflected by the Y–Ti–O particles was significantly reduced [75] due to the formation of a larger Y–Al–O (5–100 nm) phase in the ODS FeCrAl alloy containing Ti, and the number density of the Y–Ti–O phase could be neglected according to the statistical results [28,74,87,127]. Microstructure observation showed that larger Y–Al–O phases are the dominant strengthening phases, such as YalO_3_ (Yttrium Aluminum Perovskite, YAP) [106,128], Y_3_Al_5_O_12_ (Yttrium–Aluminum–Garnet, YAG) [129], and Y_4_Al_2_O_9_ (Yttrium–Aluminum–Monoclinic, YAM) [130]. Specifically, Dubiel et al. [131] confirmed that the majority of Y-Al oxides in MA956 were tetragonal Y_3_Al_5_O_12_ (Yttrium Aluminum Tetragonal, YAT). In PM2000 (Fe–20Cr–5.5Al–0.5Ti–0.5Y_2_O_3_), four Y–Al–O types of nano-oxides could be found in the matrix, which were identified as monoclinic YAM, YAT, YAG [132], and YAP [133]. These microstructural examinations above lead to the conclusion that Y–Ti oxides were not present, but instead Y–Al oxides with larger sizes were formed when ODS F/M steels contain both aluminum and titanium, which led to the reduction of the tensile and creep properties of ODS alloys [28,74].

**Figure 18:**
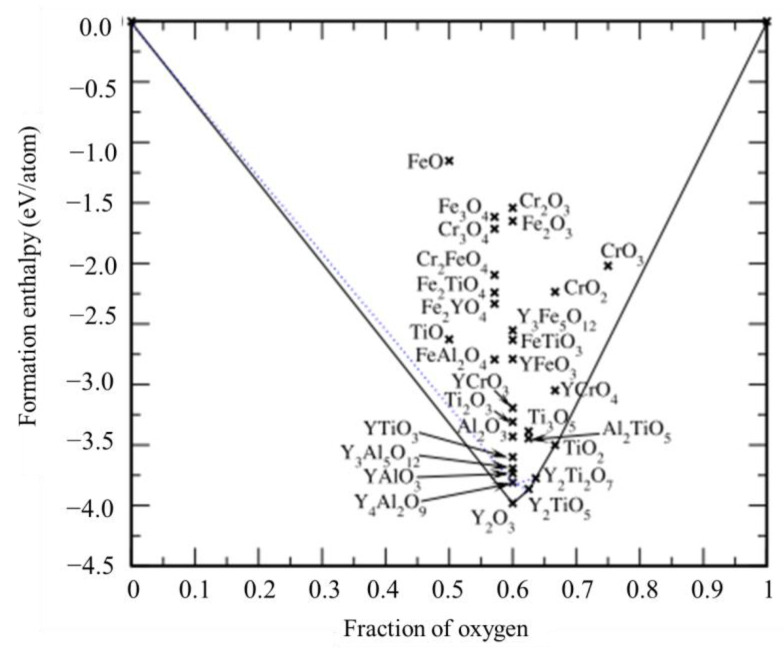
DFT-computed formation enthalpies of likely oxides of ODS steel plotted as a function of oxygen fraction. Oxides with relatively more negative formation enthalpies, found at the bottom of the figure, such as Y_2_O_3_, Y_2_TiO_5_, Y_4_Al_2_O_9_, Y_2_Ti_2_O_9_, and YAlO_3_, are actually the set of phases often observed in the microstructure of typical ODS steels [134].

In view of the formation mechanism of this phenomenon, Chinnappan [134] first used density functional theory-based calculated formation and reaction enthalpies to examine the relative stability of a large number of likely oxide phases of typical ODS steels based on the Fe–Cr–Al–Ti–Y–O system. As shown in Figure 18, Y_2_O_3_, Y_4_Al_2_O_9_, YalO_3_, Y_2_Ti_2_O_7_, and Y_2_TiO_5_ oxides with relatively more negative formation enthalpies are more stable, corroborating their observation in the microstructure of ODS steels, which indicates that they are favored over other likely oxides, such as those based on Y–Fe–O, Y–Cr–O, and Fe–Cr–O. In addition, it can also be seen from the convex–hull diagram (Figure 18) that Y–Al–O and Y–Ti–O do have a certain competitive relationship with respect to formation, which may be the reason why the strengthening effect of Ti is not significant in ODS FeCrAl alloy when the amount of Al addition (≥5 wt.% ) is much greater than that of Ti (~0.3 wt.%). However, Kamikawa et al. [135] found in the creep test for ODS FeCrAl alloy with and without Ti that the creep resistance of the ODS FeCrAl alloy with Ti was much higher than that of the model alloy without Ti, which showed that Ti plays a positive role in improving the comprehensive mechanical properties.

### 3.4. Zr

**Figure 19:**
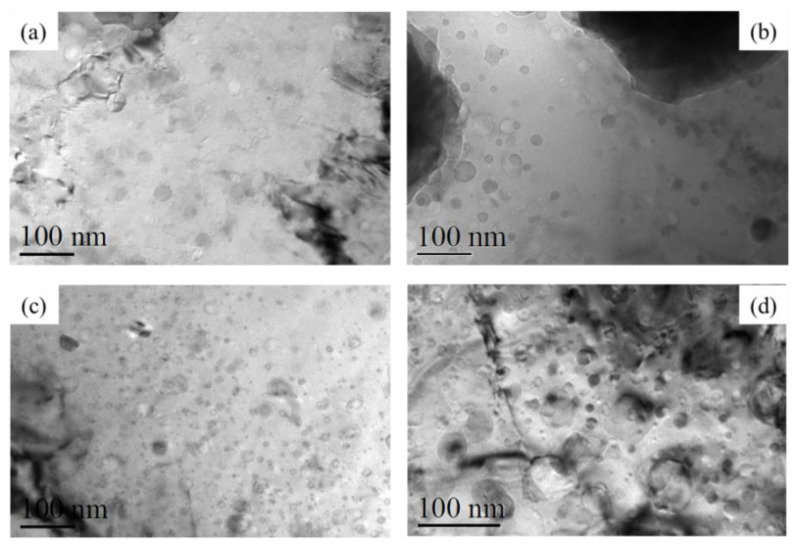

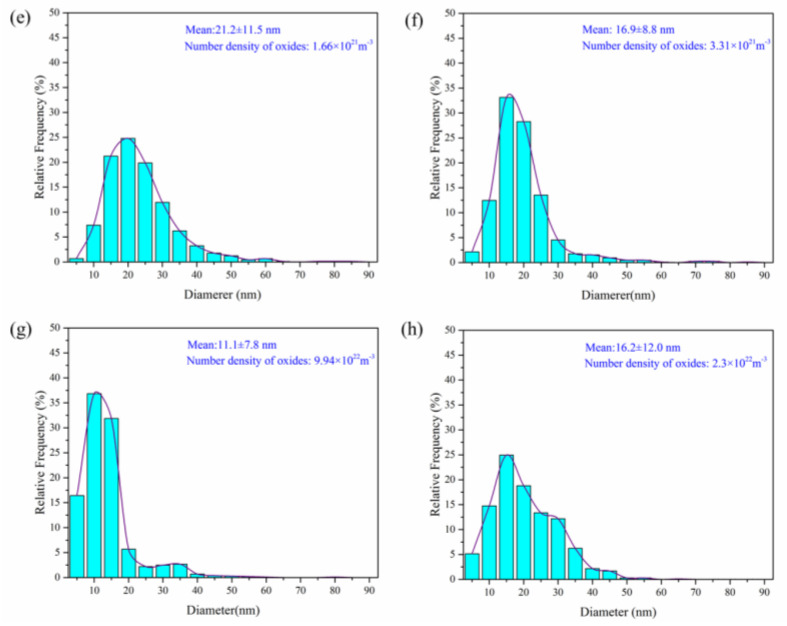
TEM images of oxides in 0Zr (Fe–12Cr–5Al–2W–0.3Y_2_O_3_–0Zr) (**a**) 0.3Zr (Fe–12Cr–5Al–2W–0.3Y_2_O_3_–0.3Zr) (**b**) 0.6Zr (Fe–12Cr–5Al–2W–0.3Y_2_O_3_–0.6Zr) (**c**), 1Zr (Fe–12Cr–5Al–2W–0.3Y_2_O_3_–1Zr) (**d**) the corresponding histograms of oxide particle size distribution of 0Zr (**e**), 0.3Zr (**f**), 0.6Zr (**g**), and 1Zr (**h**) [143].

According to the problems encountered in the development of the nuclear-level ODS FeCrAl alloys mentioned above, Kimura et al. [28] prepared a series of ODS FeCrAl ferritic steels with Zr addition by considering the lower oxide formation energy [136,137] and the diffusion rate of Zr in the bcc Fe matrix [129] to eliminate the negative effect on mechanical properties due to the Al addition. Fine Y–Zr–O oxides (<15 nm) formed in the bcc Fe matrix possess a coherence or semi–coherence relationship with the bcc matrix and endow ODS FeCrAl alloys with better corrosion resistance, mechanical properties, and radiation tolerance [138]. For instance, the corrosion resistance of the Zr–added ODS FeCrAl alloys in the coolant of supercritical water [64] and lead–bismuth eutectic [139,140] was proved to be superior compared to the Zr–free alloys due to the lower Gibbs free energy of Zr for oxide formation compared to Al, resulting in a higher Al concentration available for oxide layer formation on the surface. In the test of irradiation resistance at 773 K up to 10 dpa, the oxide particles were relatively stable, and the growth rate of the radiation-induced dislocation loops at the interface between the Y–Zr–O oxides and the matrix was low in Zr-added alloys during the electron irradiation [141]. As for the mechanical properties, the long-term creep strength of the ODS Zr–containing steel at 973 K [28] was improved markedly compared with ODS Zr–free steel, and the ultimate tensile strength (UTS) [36] was found to reach up to approximately 1400 Mpa at room temperature. Furthermore, the fracture toughness and microhardness of the ODS FeCrAl steels were also improved by adding Zr due to the increased volume fraction of fine equiaxed ferritic grains and the number density of nano-oxide particles, as well as the decreased average size of nano-oxides [142]. Dedicated research has been conducted on the effects of Zr content on microstructure and mechanical properties in the ODS FeCrAl alloy system (Fe–12Cr–5Al–2W–0.3Y_2_O_3_–xZr (x = 0, 0.3, 0.6, and 1.0 wt%)) [143], which shows that the number density and size of oxides significantly depend on Zr content due to the formation of Y–Zr–O oxides, as shown in Figure 19. This tendency is similar to the effects of Ti on ODS Fe–Cr alloys mentioned in Section 3.2. Thus, it is clear that the content optimization of oxide-forming elements can be partially determined by their influence on the microstructures. Of course, design for alloy composition should also be considered in the context of nuclear applications. For example, systematic experiments on resistance to radiation, oxidation, creep, and impact should be carried out for proposed model alloys to comprehensively evaluate and choose the optimized alloy.

**Figure 20:**
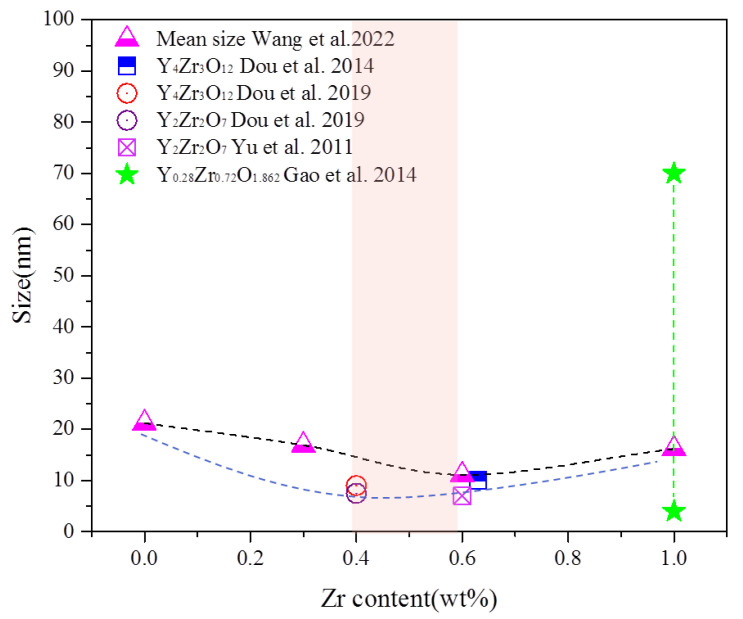
Size of the oxides dependent on Zr content [131,133,134,135,136].

Regarding the types of oxides in Zr-containing ODS FeCrAl alloys [28], the species of Y–Zr–O phases reported include Y_4_Zr_3_O_12_ [138], Y_2_Zr_2_O_7_, or Y_6_ZrO_11_ [141]. Y_0.28_Zr_0.72_O_1.862_ [144]. Specifically, Dou et al. [138] found that almost all the small nanoparticles with diameter smaller than 10 nm in SOC–14 (Fe–15Cr–2W–0.1Ti–4Al–0.63Zr–0.35Y_2_O_3_) hot extruded at 1150 °C were consistent with trigonal δ-phase Y_4_Zr_3_O_12_ oxides and coherent with the bcc steel matrix. They also [145] detected trigonal δ-phase Y_4_Zr_3_O_12_ (9 nm) and cubic Y_2_Zr_2_O_7_ (7.5 nm) coexisting in UOC–10 (Fe–15Cr–2W–0.5Ti–7Al–0.4Zr–0.5Y_2_O_3_) hot extruded at 1150 °C. Yu et al. [141] identified oxides as Y_2_Zr_2_O_7_ structures with a size of approximately 7 nm formed in Fe–16Cr–4Al–0.6Zr–0.35Y_2_O_3_ hot extruded at 1150 °C. Gao et al. [144] identified oxide particles with a size range of 4–70 nm as Y_0.28_Zr_0.72_O_1.862_ precipitated in Fe–16Cr–4Al–2W–0.5Ti–0.4Y_2_O_3_–1Zr fabricated by a sol–gel method combined with mechanical alloying and spark plasma sintering (SPS) techniques at 1200 °C. On the basis of the above data, a systematic study of the influence of Zr content on the microstructure and mechanical properties of ODS FeCrAl was conducted by Ren et al. [146] and Wang et al. [143]. The detected Zr-oxides are trigonal δ-phase Y_4_Zr_3_O_12_, and excess Zr results in coarsening of the oxide particles, indicating that the Zr content affects the trend of the oxide size distribution. This conclusion is similar to the influence of Ti contents on the size distribution of oxides. The comparison of the mean size of the oxides in alloys containing different Zr contents in previous studies is shown in Figure 20. From the perspective of minimizing the average size of oxides, it can be concluded that the content of Zr should be appropriate in the range of 0.4–0.6 wt.%. However, it should be noted that small changes in many factors, such as process and alloy composition, may affect the types of oxide in the alloy, according to the detection results in previous studies.

**Figure 21:**
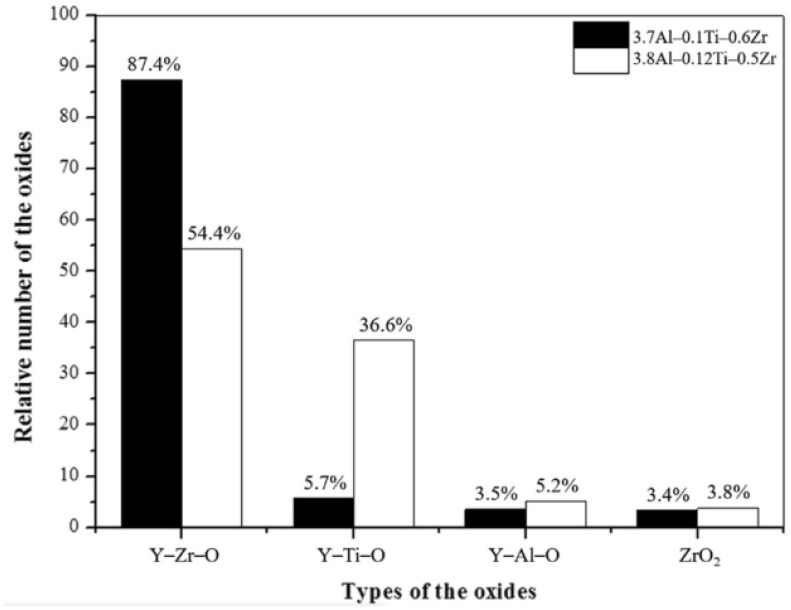
Proportions of Y–Zr, Y–Ti, and Y–Al complex oxides and ZrO_2_ [148].

As for the formation mechanism and priority of oxides, comparison between the binding energies of oxide-forming elements, such as Ti, Al, and Zr, by first-principles calculation explained the change in oxide types in ODS FeCrAl in previous studies. During MA, repeated and severe plastic deformation results in high stored energy and a high density of defects, such as vacancies and dislocations, in powders [147], which provide a great number of favorable nucleation sites for the precipitation of nano-oxides. On the basis of the first principles calculations, in all the stages of formation, Y–Zr–O vacancy clusters (−4.16 eV) have higher binding energy than Y–Al–O vacancy clusters (−3.25 eV) in the iron matrix of bcc, which implies that in a ferritic steel both containing Zr and Al, the Y–Zr–O clusters are more stable and are favored to nucleate over the Y–Al–O clusters [136]. Therefore, for Zr-added ODS FeCrAl alloys, Y–Zr–O is the main strengthening phase, with less Y–Al–O co-existing in the matrix [148,149]. Regarding ODS FeCrAl alloys added with Ti and Zr, Dou et al. [148] found that Y–Zr–O, Y–Al–O, and Y–Ti–O oxides co-existed in the matrix in different proportions by HRTEM identification and statistics. As shown in Figure 21, from the experimental results, we can see that the affinity of Zr, Al, Ti, and Y_2_O_3_ is Zr > Ti > Al. This experimental phenomenon is consistent with the calculation results of Qian et al. [137] using the first principle. The simultaneous precipitation of Y–Zr–O, Y–Al–O, and Y–Ti–O nano-oxides in ODS FeCrAl may bring some combined benefits to the ODS FeCrAl alloy, but there are still many uncertainties, such as the possible complex interactions between these nanoprecipitates and their potential impact on performance. In addition, density functional theory-based calculations of formation and reaction enthalpies [134] examined the relative stability of a large number of likely oxide phases of typical ODS FeCrAl alloys based on the Fe–Cr–Al–Zr–Y–O system, with the Fe–Cr–Al–Zr–Ti–Y–O system still to be performed.

**Figure 22:**
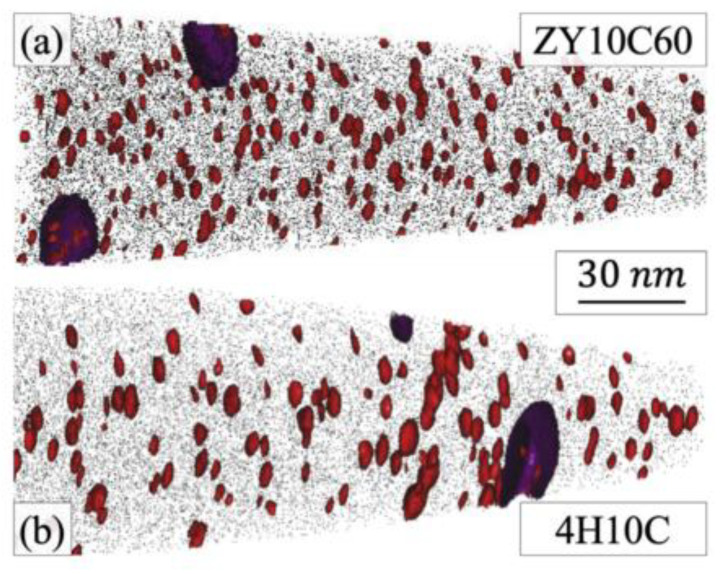
APT control volumes for CrAZY (Fe–10Cr–6.1Al–0.3Zr + 0.3Y_2_O_3_) (**a**) powder ZY10C60 (Fe–10Cr–6.1Al–0.3Zr + 0.3Y_2_O_3_) annealed for 1 h at 1000 °C and (**b**) alloy 4H10C (Fe–10Cr–6.1Al–0.3Zr + 0.3Y_2_O_3_) annealed for 1 h at 1000 °C followed by extrusion. Depicted are red 1.5 at.% (Y, Al, and O) isoconcentration surfaces and purple 10 at.% Zr isoconcentration surfaces atop 0.1% of black Fe matrix atoms [151].

**Figure 23:**
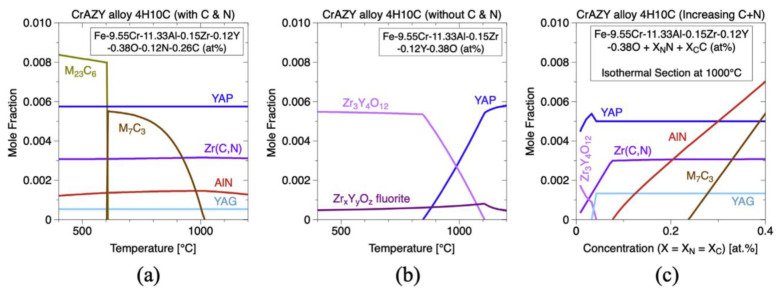
Thermodynamic calculation results for expected CrAZY alloy phases in the 4H10C alloy (**a**) with and (**b**) without C and N impurity elements. Additionally, shown in (**c**) is the effect of increasing C and N concentrations on the equilibrium phases at 1000 °C [151].

Noting the 2nd generation ODS FeCrAl alloy (Fe–10Cr–6.1Al–0.3Zr–0.3Y_2_O_3_) developed recently at the Oak Ridge National Laboratory [150,151,152], two different types of precipitates form in the alloy system, which has a high number density (>10^23^ m^−3^) of (Y, Al, and O)-rich nano-oxides (~2–4 nm diameter). As shown in Figure 22, the nanoprecipitate population is comprised of a dispersion of red precipitates highlighted using Y-, YO-, and AlO-rich isoconcentration surfaces. In addition, coarser precipitates enriched in Zr elements are highlighted in purple, but no Y–Zr–O phases are found in the matrix. Massey et al. [151] carried out a thermodynamic assessment of Zr precipitation and found that (C,N) solute impurities significantly affect the equilibrium phases within the alloy system. As shown in Figure 23a, when C and N exist in the matrix, the primary phases that are expected to form are Zr(C,N), AlN, Cr carbides, and complex (Y, Al, and O)-rich oxides, such as YAP and YAG. However, when C and N are removed completely from the alloy system (Figure 23b), the high affinity of both Zr and Al for O in solution leads to a competition for the formation of Zr_3_Y_4_O_12_ and YAP (YAlO_3_) at the annealing/extrusion temperature of 1000 °C. In other work by Massey et al. [126], this concept of impurity sequestration was used to optimize the alloy composition and microstructure.

**Figure 24:**
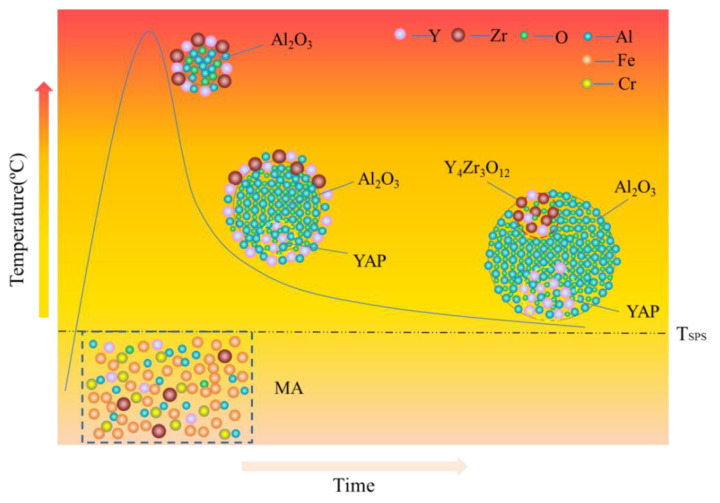
Schematic diagram for the formation of appendage oxide [143].

In addition, an interesting phenomenon has been found in previous studies of ODS FeCrAl alloys, called co-nucleation or co-precipitation of oxides [129] or appendage oxide [143,153] with multiple phases. Unocic et al. [129] found co-nucleation of different oxide phases in their model alloys containing Al elements with different powders of ZrO_2_, HfO_2_, and Y_2_O_3_, respectively, e.g., Y_3_Al_5_O_12_/Al_2_O_3_ in 125Y alloy, Zr(C,N)/Al_2_O_3_ and Zr(C,N)/Y_3_Al_5_O_12_ in 125YZ alloy, HfO_2_/Al_2_O_3_, Y_2_Hf_2_O_7_/Al_2_O_3_ and complex Y_3_Al_5_O_12_/Y_2_Hf_2_O_7_/Hf(C,N) or Al_2_O_3_/Y_2_Hf_2_O_7_/Hf(C,N) in 125YH alloy produced by high kinetic energy ball milling and hot extrusion. Klimiankou et al. [154,155] found co-nucleation of complex Y–Al–O/Al_2_O_3_, i.e., two or more smaller Y–Al–O fractions located on the significantly larger α-Al_2_O_3_ fraction, and Y–Al–O/TiC, i.e., various Y–Al–O particles trapped on the surface or even in the center of a large Ti(C,N) inclusion in PM2000. Zhou et al. [156] found three phenomena in HRTEM: (1) one Y_2_O_3_ particle containing two YAM particles; (2) an oxide particle containing YAG and Y_2_O_3_ simultaneously; and (3) the interaction between YAG and YAM. Shi et al. [153] found coarse complex Al_2_O_3_/Y_4_Zr_3_O_12_ oxides, which were defined as “appendage oxides”, distributing in the matrix of an as-deposited sample prepared by laser-engineered net-shaped (LENS) technology. A co-precipitation consisting of ZrC and Al_2_O_3_ with Y segregation to the surface of the alumina precipitate was discovered in ZY10C60 annealed powder [151]. Nanoscale Y_4_Zr_3_O_12_/YAP/Al_2_O_3_ appendage oxides [143] were recently found in the Fe–12Cr–5Al–2W–1Zr model alloy prepared by SPS. Table 3 summarizes the types of co-precipitations in previous studies. It should be noted that the formation mechanism of oxides with multiple phases in Al-containing ODS alloys might be significantly influenced by various hot consolidation processes, such as hot extrusion (HE) [129], hot isostatic pressing (HIP) [154,155], spark plasma sintering (SPS) [143,155], as well as laser additive manufacturing technology [153], due to different thermal histories. Each preparation process can form a thermal history with its own characteristics; for example, in the process of SPS, the formation of a liquid sintering neck may lead to the formation of α-Al_2_O_3_/YAP/Y_4_Zr_3_O_12_ complex oxides, as shown schematically in Figure 24 [143]. However, most co-precipitations were formed under solid-state conditions; thus, the preferential formation mechanism in thermodynamics should be the main reason for this phenomenon. For example, in annealed powder [151], co-precipitation of ZrC/Al_2_O_3_/Y strongly suggests that when interstitial elements, such as C and N, are present in the alloy system, Zr will preferentially react with these elements instead of competing with Al for the formation of (Zr, Y, and O)-rich oxides, which is consistent with the results of the thermodynamic analysis, as shown in Figure 24.

The discovery of “core/shell structure” captured the evolution process of the precipitation phase in ODS alloys and clearly indicated that there is a possible competitive relationship between some elements. In previous studies, for ODS Al-free Fe–Cr alloys with Ti, the core/shell structures included (Y, Ti, and O)-rich core/Cr shell, i.e., Cr-rich shell encircling the smallest (Y, Ti, and O)-rich nano-precipitates [157,158,159,160] and (Y, Zr, and O)-rich oxide core surrounded by Ti-rich shell were found in Al-free ODS Fe–Cr alloy containing both Ti and Zr at 1000 °C [161]. Two hypotheses for the phenomenon of the “core/shell structure” were proposed: the first hypothesis is that the shell is a consequence of segregation at the interface between the particle and matrix after the particle has formed. The second theory is that the shell could be essential for the precipitation of the particle and may play the role of an interfacial phase, lowering the surface energy of the particle and thus promoting the particle to precipitate [162]. The results of the first principal calculation reasonably explained the formation of Y, Ti, and O core/Cr shells in Al-free ODS Fe–Cr alloys containing Ti. For instance, interactions among the Y, Ti, and O and vacancy were compared by Murali et al. [163], Cr is showing negligible interaction with vacancy, O, and O–V and interacts repulsively with Y and Ti, resulting in the “core/shell structure” for the nanoclusters consisting of a (Y, Ti, and O)-rich core and a Cr shell. As for the intrinsic cause of the formation of the (Y, Zr, and O)-rich core/Ti-rich shell structure [161], the binding energies of Zr with Y, O, and V and their clusters in bcc Fe (Ti was replaced with Zr in the defect cluster) were calculated by Murali et al. [163]. The research results indicated that Zr interacts attractively with Y as compared with the repulsive interaction of Ti with Y. In addition, when Zr takes the place of Ti, a higher binding energy of O, V, and their clusters is displayed. Furthermore, the binding energies of Y–V–O–Zr were higher compared with the Y–V–O–Ti clusters in all the different configurations studied, indicating the clusters have higher thermal stability. In the minimum energy configuration of Y–V–O–Zr, the bond lengths of Y–O and Zr–O were 2.30 Å and 2.05 Å, respectively; however, in the Y–V–O–Ti defect cluster, the bond lengths of Y–O and Ti–O were 2.71 Å and 2.37 Å, respectively. Thus, supposing that Y, V, O, Zr, and Ti are uniformly distributed in the mechanically alloyed supersaturated powder, once the reaction conditions are appropriate, the stronger attraction of Zr elements to Y, V, and O is likely to extrude Ti elements to form a Ti-rich region around them, which leads to the generation of a core–shell structure.

**Figure 25:**
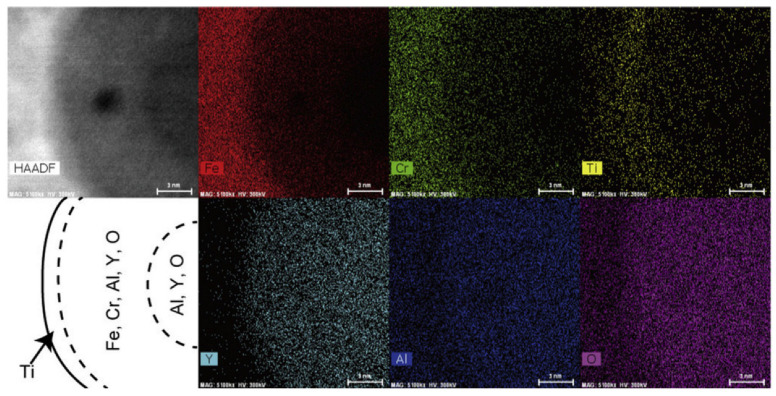
Magnified images of a coarse oxide particle in 15Cr–7Al after annealing at 1623 K for 27 h. The scale bars measure 3 nm [164].

**Figure 26:**
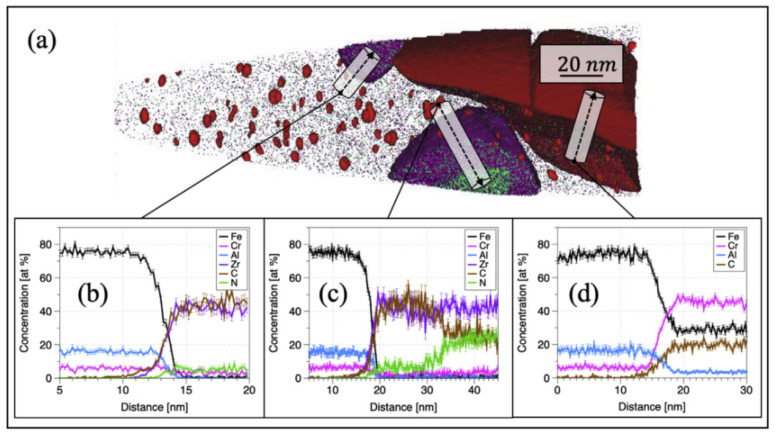
An (**a**) APT control volume of ZY10C60 annealed powder with multiple types of precipitates atop black Fe matrix atoms. Red 1.5 at.% (Y, Al, and O) isoconcentration surfaces highlight the smallest precipitate population, while brown 20 at.% Cr isoconcentration surfaces and purple 10 at.% Zr isoconcentration surfaces show larger precipitates in the volume. One-dimensional concentration profiles through larger precipitates provide evidence for (**b**) ZrC, (**c**) Zr(C,N), and (**d**) (Fe,Cr) 23C6. (For interpretation of the references to color in this figure legend, the reader is referred to the Web version of this article) [151].

**Figure 27:**
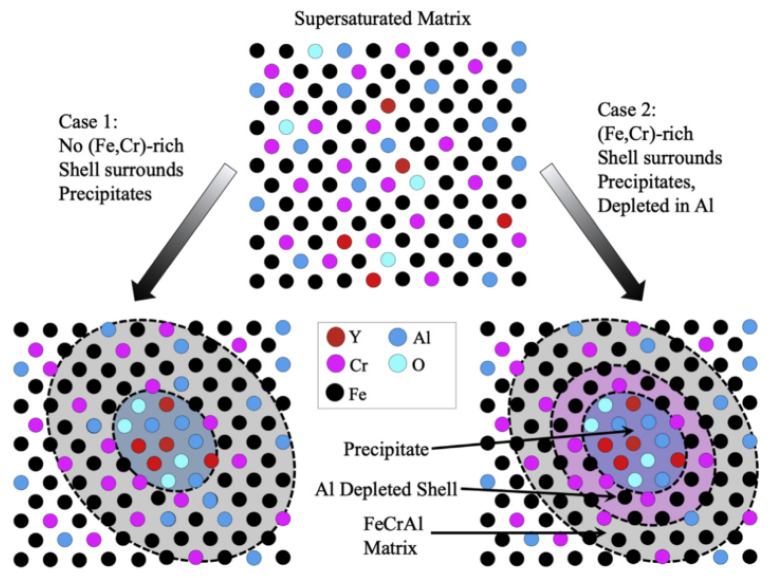
Visualization of the two limiting cases for precipitate compositional correction [151].

With respect to ODS FeCrAl alloys, the core/shell structures found include the Y–Al–O core/Ti shell [164] and Zr(C,N) core/ZrC shell [151]. For instance, Oono et al. [164] discovered that a large oxide particle in 15Cr–7Al annealed at 1623 K for 27 h had a core–shell structure; the core contained Al, Y, and O, while the shell additionally contained Fe and Cr. Ti was distributed on the surface of the shell (see Figure 25). Monoclinic Y_4_Al_2_O_9_ (YAM) or orthorhombic YAlO_3_ (YAP) were indexed in 15Cr–7Al [164]. Although we cannot judge the possible cause of this phenomenon at will, it could be inferred from the existing thermodynamic calculation results shown in Figure 18 that in the alloy system with Al and Ti, there might be multiple Y–Ti–O phases produced during the preparation. If there exists a kind of Y–Ti–O phase less stable than the Y–Al–O phase, such as YTiO_3_, which may lead to the above phenomenon occasionally or frequently. The finding of Zr(C,N) core/ZrC shell (see Figure 26) indicates that under certain conditions, Zr(C,N) should be a relatively more stable phase in thermodynamics compared with ZrC [151]. In addition, Massey et al. [151] assumed a new type of model of Y–Al–O core/(Fe,Cr)-rich shell structure, as shown in Figure 27. Although this phenomenon was not found in the experiment, it should exist in a specific alloy system theoretically, and more work in characterization may be required to prove its existence.

### 3.5. W

According to the design requirements of reduced-activation ODS steels, alloying elements with a slow decay rate, such as Mo and Nb in conventional steels, are substituted by elements with a higher decay rate of radioactivity, such as W, V, and Ti. Another reason for this approach is that the strengthening function of Mo and Nb in steels could be compensated by W, V, and Ti [165,166]. Tungsten (W) as the ferrite forming element was first introduced into 9 Cr low-activation steel for fusion reactors as a solution-strengthened alloy element [167], which improved creep fracture time at 873 K (600 °C) of 9 Cr low-activation steel. Similarly, compared with the MA957 alloy containing Mo, the creep properties of the ODS ferritic alloy containing W, such as 12YWT, have been significantly improved up to 850 °C [99,100,168,169]. To explore the effect of W on the microstructure, the model alloys Fe–12.4Cr–0.25Y_2_O_3_ (12CrY) ODS and 12CrYW were developed by ORNL of the United States. By comparing with 12CrY (cluster size 10–40 nm, 10^20^–10^21^ m^−3^) alloy, W in 12CrYW can increase the number density (3.9 × 10^23^ m^−3^) and reduce the size of clusters in the absence of Ti [170]. Dou et al. [171] also suggested that although W has less effect on the microstructure than oxide-forming elements such as O and Ti, it still plays a positive role in reducing the size and increasing the density of oxides. Furthermore, W can also improve the thermal stability and corrosion resistance of ODS alloys [172]. In terms of mechanical properties, Japanese scholars [173] have tested the effect of W content (nominal content is 2.0 wt.%, 2.5 wt.%, 3.0 wt.%, and 3.5 wt.%) on 9Cr–xWVTa steels. The results showed that when the W content was increased from 2.0 wt.% to 2.5 wt.%, the strength increased and the total elongation of the alloy decreased slightly. When it was further increased to 3.5%, it hardly changed. The laves phase due to excessive addition of the element W is the main reason for the reduction of properties. Therefore, it is appropriate to control the content of W within 2%.

In conclusion, through the summary and induction of the alloy elements, it is found that each element not only plays the role and influence of itself in the alloy but also that there is a complementary or competitive relationship between the elements. These factors form the complexity of the nuclear ODS FeCrAl alloy system, and they can also provide a basis for reasonable design of the ODS FeCrAl alloy.

## 4. Conclusions

The research and development of ODS FeCrAl is a complex and systematic project. This article reviews previous research findings and makes summarized statements on the following aspects:

(1) The effects of Cr and Al content in the ODS FeCrAl alloy on the corrosion behavior, radiation, thermal stability of the matrix, and processing feasibility were summarized, and the range of reasonable design content of the two elements above was stated in detail;
(2) The role of oxide-forming elements, i.e., Y (Y_2_O_3_), Ti, Al, Zr, and solid solution strengthening elements, i.e., W, in ODS FeCrAl alloy and the design basis for their reasonable content were concluded;
(3) The important effects of different alloy elements on the type and size distribution of the oxide particles were introduced;
(4) The density functional theory-based calculation of formation and reaction enthalpies to examine the relative stability of a large number of likely oxide phases of typical ODS FeCrAl alloys based on the Fe–Cr–Al–Zr–Y–O system, with the Fe–Cr–Al–Zr–Ti–Y–O system still to be performed;
(5) In the as-prepared ODS FeCrAl alloy, several populations of nano-oxides can precipitate simultaneously. However, in the process of preparing cladding tubes, due to the generation of a large number of dislocations, these oxide-forming elements may diffuse along the dislocation pipes due to the interaction between the oxides and dislocations. During this process, oxides may undergo the phenomenon of “dissolution–precipitation–re-dissolution–re-precipitation”. These types of oxides may even undergo chemical reactions to generate non-equilibrium products. These possible changes in the microstructure caused by the competition between the various alloying elements, as well as their impacts on the service performance of the ODS FeCrAl alloy, should be paid attention to in future research;
(6) Based on the review, several other aspects of the ODS FeCrAl alloy will be reviewed in another paper, such as the effects of heat solidification parameters on the microstructure and mechanical properties; and a comparison of the high temperature stability and irradiation stability of typical oxides, i.e., Y–Ti–O, Y–Al–O, and Y–Zr–O phases; and a summary of the microstructure evolution of ODS FeCrAl alloy under plastic deformation. These will contribute to a comprehensive understanding of the entire alloy development process.

## Figures and Tables

**Table 1:** Non-irradiation oxidation experiment data at ultra-high temperature (T > 1000 °C) simulated accident conditions.

C ^1^ (wt.%)	Experimental Condition			CP ^5^	OLT ^6^ (μm)	Refs.
	T ^2^ (°C)	T ^3^ (h)	At ^4^			
SUS430 (Commercial Fe–Cr alloy)	1050	200	air	Fe, Cr, and Mn phases	20	[54]
S1(Fe–16Cr–4Al–0.1Ti–0.35Y_2_O_3_)	1050	200	air	α-Al_2_O_3_	3.5	[54]
Fe–14Cr–4.5Al–0.35Ti–2W–0.6Y_2_Ti_2_O_7_	1100	200	air	α-Al_2_O_3_	4	[55]
125YZ	1200	4	air	α-Al_2_O_3_	2.41 ± 0.2	[58,59]
			steam		2.34 ± 0.1	
106ZY-40 h	1200	4	air	α-Al_2_O_3_	slightly thinner than 125YZ	[60]
			steam			
15Cr–7Al–0.5Ti–0.5Y_2_O_3_–xZr–yEx.O x = 0~0.6 y = 0.09~0.22	1200	~25	air steam	α-Al_2_O_3_	--	[52]
	1300	~25			--	
	1400	~25			~7 *	
	1500	~0.5			totally oxidized	
FeCrAlZr–ODS	1450	25	UO_2_ inert gas	α-Al_2_O_3_	3.6	[53]
	1621	--	steam	U-O-Al-Fe-Cr	totally oxidized	

^1^ Composition, ^2^ Temperature, ^3^ Time, ^4^ Atmosphere, ^5^ Corrosion products, ^6^ Oxide layer thickness, * Estimated according to pictures in the literature.

**Table 2:** Non-irradiation oxidation experiment data in SCW at operating temperature (T < 1000 °C).

Composition (wt.%)	Experimental Condition			Corrosion Products	Thickness (μm)	Ref.
	Temperature (°C)	Time (h)	Environment			
430 SS (Commercial Fe–Cr alloy)	500	1000	SCW	Outermost: Fe_2_O_3_ Outer: Fe_3_O_4_ Inner: (Fe,Cr)_3_O_4_	≥15	[64]
16Cr–4Al	550	250	SCW	Outer: hematite, magnetite Inner: Al_2_O_3_	0.1	[63]
16Cr–3Al (Fe–15.98Cr–2.64Al–0.42Zr–0.29Y_2_O_3_)	600	200	SCW	Outer: Hexagonal (Cr, Fe)_2_O_3_ Inner: Al_2_O_3_	0.25	[65]
		400			--	
		600			0.35	
		800			--	
		1000			0.4	
SOC–1 (Fe–16.11Cr–3.44Al–0.09Ti–0.34Y_2_O_3_)	400	8760 (1Y)	SCW	Outermost: Fe_2_O_3_ Outer: Al_2_O_3_, Inner: (Fe,Cr)_3_O_4_	--	[64]
	500				--	
	600				5	

**Table 3:** Types of co-precipitations containing different phases.

Alloy (wt.%)	Consolidation/Heat Treatment	Co-Precipitations	Refs.
125Y (FeCrAl–Y_2_O_3_)	Mechanical milling hot extrusion heat treated at 950 °C for 1 h	Y_3_Al_5_O_12_/Al_2_O_3_	[129]
125YZ (FeCrAl–Y_2_O_3_–HfO_2_)		Zr(C,N)/Al_2_O_3_, Zr(C,N)/Y_3_Al_5_O_12_	
125YH (FeCrAl–Y_2_O_3_–ZrO_2_)		HfO_2_/Al_2_O_3_, Y_2_Hf_2_O_7_/Al_2_O_3_, Y_3_Al_5_O_12_/Y_2_Hf_2_O_7_/Hf(C,N), Al_2_O_3_/Y_2_Hf_2_O_7_/Hf(C,N)	
PM2000 (Fe–19Cr–5.5Al–0.5Ti–0.5Y_2_O_3_)	--------	Y–Al–O/Al_2_O_3_ Y–Al–O/TiC	[154,155]
Fe–9Cr–0.1C–2W–0.2V–0.07Ta–0.05Al–0.35Y_2_O_3_ Fe–9Cr–0.1C–2W–0.2V–0.07Ta–0.1Al–0.35Y_2_O_3_	Mechanical milling SPS annealed at 800 °C for 1 h	Y_2_O_3_/YAM YAG/Y_2_O_3_ YAG/YAM	[156]
Fe–15Cr–2W–4.5Al–0.3Ti–0.3Zr–0.3Y–0.2Y_2_O_3_	Mechanical milling laser-engineered net-shaped (LENS) technology	Al_2_O_3_/Y_4_Zr_3_O_12_	[153]
CrAZY alloy (Fe–10Cr–6.1Al–0.3Zr + 0.3Y_2_O_3_)	Mechanical milling hot extrusion	ZrC/Al_2_O_3_/Y	[151]
Fe–12Cr–5Al–2W–xZr (1Zr)	Mechanical milling SPS	Y_4_Zr_3_O_12_/YAP/Al_2_O_3_	[143]

## Data Availability

Data availability is not applicable to this article as no new data were created or analyzed in this review.

## References

[1] Zinkle S.J., Terrani K.A., Gehin J.C., Ott L.J., Snead L.L. (2014). Accident tolerant fuels for LWRs: A perspective. J. Nucl. Mater..

[2] Department of Energy (2015). Development of Light Water Reactor Fuels with Enhanced Accident Tolerance. Report to Congress.

[3] Rebak R.B., Andresen P.L., Kim Y.J., Dolley E.J. (2014). Characterization of Advanced Steels as Accident Tolerant Fuel Cladding for Light Water Reactors.

[4] Rebak R.B. Characterization of Advanced Steels as Accident Tolerant Cladding for Light Water Reactor Nuclear Fuel. Proceedings of the ASME 2015 Pressure Vessels and Piping Conference.

[5] Rebak R.B. (2015). Alloy selection for accident tolerant fuel cladding in commercial light water reactors. Metall. Mater. Trans. E.

[6] Rebak R.B., Terrani K.A., Fawcett R.M. FeCrAl Alloys for Accident Tolerant Fuel Cladding in Light Water Reactors. Proceedings of the ASME 2016 Pressure Vessel & Piping Conference.

[7] Rebak R.B., Larsen M., Kim Y.J. (2017). Characterization of oxides formed on iron-chromium-aluminum alloy in simulated light water reactor environments. Corros. Rev..

[8] Rebak R.B., Terrani K.A., Gassmann W.P., Williams J.B., Ledford K.L. (2017). Improving nuclear power plant safety with FeCrAl alloy fuel cladding. MRS Adv..

[9] Dolley E.J., Schuster M., Crawford C., Rebak R.B. (2018). Mechanical behavior of FeCrAl and other alloys following exposure to LOCA conditions plus quenching. Proceedings of the 18th International Conference on Environmental Degradation of Materials in Nuclear Power Systems—Water Reactors.

[10] Tortorelli P.F., Brady M.P. (2000). Alloy design approaches for high-temperature oxidation resistance. JOM.

[11] Terrani K.A. (2018). Accident tolerant fuel cladding development: Promise, status, and challenges. J. Nucl. Mater..

[12] Opila E.J., Jacobson N.S., Myers D.L., Copland E.H. (2006). Predicting oxide stability in high-temperature water vapor. JOM.

[13] Cheng T., Keiser J.R., Brady M.P., Terrani K.A., Pint B.A. (2012). Oxidation of fuel cladding candidate materials in steam environments at high temperature and pressure. J. Nucl. Mater..

[14] Miller M.K., Russell K.F., Hoelzer D.T. (2006). Characterization of precipitates in MA/ODS ferritic alloys. J. Nucl. Mater..

[15] Alinger M.J., Odette G.R., Hoelzer D.T. (2009). On the role of alloy composition and processing parameters in nanocluster formation and dispersion strengthening in nanostuctured ferritic alloys. Acta Mater..

[16] Miller M.K., Reinhard D., Larson D.J. (2015). Detection and quantification of solute clusters in a nanostructured ferritic alloy. J. Nucl. Mater..

[17] Ohtsuka S., Ukai S., Fujiwara M. (2006). Nano-mesoscopic structural control in 9CrODS ferritic/martensitic steels. J. Nucl. Mater..

[18] Odette G.R., Alinger M.J., Wirth B.D. (2008). Recent Developments in Irradiation-Resistant Steels. Annu. Rev. Mater. Res..

[19] Ukai S., Mizuta S., Fujiwara M., Okuda T., Kobayashi T. (2002). Development of 9Cr-ODS martensitic steel claddings for fuel pins by means of Ferrite to Austenite phase transformation. J. Nucl. Sci. Technol..

[20] Ohtsuka S., Shizukawa Y., Tanno T., Imagawa Y., Hashidate R., Yano Y., Onizawa T., Kaito T., Ohnuma M., Mitsuhara M. (2023). High-temperature creep properties of 9Cr- ODS tempered martensitic steel and quantitative correlation with its nanometer- scale structure. J. Nucl. Sci. Technol..

[21] Zinkle S.J., Boutard J.L., Hoelzer D.T., Kimura A., Lindau R., Odette G.R., Rieth M., Tan L., Tanigawa H. (2017). Development of next generation tempered and ODS reduced activation ferritic/martensitic steels for fusion energy applications. Nucl. Fusion.

[22] Odette G.R. (2018). On the status and prospects for nanostructured ferritic alloys for nuclear fission and fusion application with emphasis on the underlying science. Scr. Mater..

[23] Wharry J.P., Swenson M.J., Yano K.H. (2017). A review of the irradiation evolution of dispersed oxide nanoparticles in the b. c. c. Fe-Cr system: Current understanding and future directions. J. Nucl. Mater..

[24] Azeem M.M., Li Z., Wang Q., Zubair M. (2019). Molecular dynamics studies and irradiation effects in ODSS alloys. Int. J. Nucl. Energy Sci. Technol..

[25] Mustafa Azeem M., Wang Q.Y., Li Z.Y., Zhang Y. (2020). Dislocation-oxide interaction in Y_2_O_3_ embedded Fe: A molecular dynamics simulation study. Nucl. Eng. Technol..

[26] Locatelli G., Mancini M., Todeschini N. (2013). Generation IV nuclear reactors: Current status and future prospects. Energy Policy.

[27] Abram T., Ion S. (2008). Generation–IV nuclear power: A review of the state of the science. Energy Policy.

[28] Kimura A., Kasada R., Iwata N., Kishimoto H., Zhang C.H., Isselin J., Dou P., Lee J.H., Muthukumar N., Okuda T. (2011). Development of Al added high—Cr ODS steels for fuel cladding of next generation nuclear systems. J. Nucl. Mater..

[29] Pint B.A., Wright I.G. (2002). Long-term high temperature oxidation behavior of ODS ferritics. J. Nucl. Mater..

[30] Pint B.A., Wright I.G. (2005). Oxidation behavior of ODS Fe-Cr alloys. Oxid. Met..

[31] Kimura A., Cho H.S., Toda N., Kasada R., Yutani K., Kishimoto H., Iwata N., Ukai S., Fujiwara M. (2007). High Burnup Fuel Cladding Materials R&D for Advanced Nuclear Systems: Nano-sized oxide dispersion strengthening steels. J. Nucl. Sci. Technol..

[32] Cho H.S., Kimura A. (2007). Corrosion resistance of high-Cr oxide dispersion strengthened ferritic steels in super-critical pressurized water. J. Nucl. Mater..

[33] Capdevila C., Miller M.K., Chao J. (2012). Phase separation kinetics in a Fe-Cr-Al alloy. Acta Mater..

[34] Capdevila C., Miller M.K., Toda I., Chao J. (2010). Influence of the a-a’phase separation on the tensile properties of Fe-base ODS PM2000 alloy. Mater. Sci. Eng..

[35] Terada M., Hupalo M.F., Costa I., Padilha A.F. (2008). Effect of alpha prime due to 475 °C aging on fracture behavior and corrosion resistance of DIN 1.4575 and MA 956 high performance ferritic stainless steels. J. Mater. Sci..

[36] Dryepondt S., Unocic K.A., Hoelzer D.T., Massey C.P., Pint B.A. (2018). Development of low-Cr ODS FeCrAl alloys for accident-tolerant fuel. J. Nucl. Mater..

[37] Lee J.S., Jang C.H., Kim I.S., Kimura A. (2007). Embrittlement and hardening during thermal aging of high Cr oxide dispersion strengthened alloys. J. Nucl. Mater..

[38] Field K.G., Hu X., Littrell K.C., Yamamoto Y., Snead L.L. (2015). Radiation tolerance of neutron-irradiated model Fe-Cr-Al alloys. J. Nucl. Mater..

[39] Briggs S.A., Edmondson P.D., Littrell K.C., Yamamoto Y., Howard R.H., Daily C.R., Terrani K.A., Sridharan K., Field K.G. (2017). A combined APT and SANS investigation of a’ phase precipitation in neutron-irradiated model FeCrAl alloys. Acta Mater..

[40] Li S., Zhou Z., Jang J., Wang M., Hu H., Sun H., Zou L., Zhang G., Zhang L. (2014). The influence of Cr content on the mechanical properties of ODS ferritic steels. J. Nucl. Mater..

[41] Noh S., Choi J.E., Choi B.K., Kang S.H., Kim T.K. (2014). Effects of Cr, Mo, Al, Zr, Y_2_O_3_ on the microstructures and tensile properties of ODS ferritic/martensitic alloys. J. Met. Mater..

[42] Bachhav M., Odette G.R., Marquis E.A. (2014). Microstructural Changes in a Neutron-Irradiated Fe-15 at. % Cr Alloy. J. Nucl. Mater..

[43] Pawel J.E., Rowcliffe A.F., Lucas G.E., Zinkle S.J. (1996). Irradiation Performance of Stainless Steels for ITER Application. J. Nucl. Mater..

[44] Tanigawa H., Shiba K., Möslang A., Stoller R.E., Lindau R., Sokolov M.A., Odette G.R., Kurtz R.J., Jitsukawa S. (2011). Status and key issues of reduced activation ferritic/martensitic steels as the structural material for a DEMO blanket. J. Nucl. Mater..

[45] Niu Y., Wang S., Gao F., Zhang Z.G., Gesmundo F. (2008). The nature of the third- element effect in the oxidation of Fe-xCr-3at.%Al alloys in 1 atm O_2_ at 1000 °C. Corros. Sci..

[46] Stott F.H., Wood G.C., Stringer J. (1995). The Influence of Alloying Elements on the Development and Maintenance of Protective Scales. Oxid. Met..

[47] Kobayashi S., Takasugi T. (2010). Mapping of 475 °C embrittlement in ferritic Fe–Cr–Al alloys. Scr. Mater..

[48] Li W., Lu S., Hu Q.M., Mao H.H., Johansson B., Vitos L. (2013). The effect of Al on the 475 °C embrittlement of Fe-Cr alloys. Comp. Mater. Sci..

[49] Han W.T., Yabuuchi K., Kimura A., Ukai S., Oono N., Kaito T., Torimaru T., Hayashi S. (2016). Effect of Cr/Al contents on the 475 °C age-hardening in oxide dispersion strengthened ferritic steels. Nucl. Mater. Energy.

[50] Field K.G., Littrell K.C., Briggs S.A. (2018). Precipitation of α′ in neutron irradiated commercial FeCrAl alloys. Scr. Mater..

[51] Yang Z., Wang Z.X., Xia C.H., Ouyang M.H., Peng J.C., Zhang H.W., Xiao X.S. (2020). Aluminum suppression of α′ precipitate in model Fe–Cr–Al alloys during long-term aging at 475 °C. Mater. Sci. Eng. A.

[52] Maeda T., Ukai S., Hayashi S., Oono N., Shizukawa Y., Sakamoto K. (2019). Effects of zirconium and oxygen on the oxidation of FeCrAl-ODS alloys under air and steam conditions up to 1500 °C. J. Nucl. Mater..

[53] Sakamoto K., Miura Y., Ukai S., Oono N.H., Kimura A., Yamaji A., Kusagaya K., Takano S., Kondo T., Ikegawa T. (2021). Development of accident tolerant FeCrAl-ODS fuel cladding for BWRs in Japan. J. Nucl. Mater..

[54] Liu T., Wang C.X., Shen H.L., Chou W.S., Iwata N.Y., Kimura A. (2013). The effects of Cr and Al concentrations on the oxidation behavior of oxide dispersion strengthened ferritic alloys. Corros. Sci..

[55] Liu T., Wang L.B., Wang C.X., Shen H.L. (2016). Effect of Al content on the oxidation behavior of Y_2_Ti_2_O_7_-dispersed Fe-14Cr ferritic alloys. Corros Sci..

[56] Weisenburger A., Jianu A., Doyle S., Bruns M., Fetzer R., Heinzel A., Del Giacco M., An W., Muller G. (2013). Oxide scales formed on Fe—Cr—Al—Based model alloys exposed to oxygen containing molten lead. J. Nucl. Mater..

[57] Tomaszewicz P., Wallwork G.R. (1983). The oxidation of high-purity iron-chromium-aluminum alloys at 800 °C. Oxid. Met..

[58] Pint B.A., Dryepondt S., Unocic K.A., Hoelzer D.T. (2014). Development of ODS FeCrAl for compatibility in fusion and fission energy applications. JOM.

[59] Unocic K.A., Hoelzer D.T., Pint B.A. (2015). Microstructure and environmental resistance of low Cr ODS FeCrAl. Mater. High Temp..

[60] Dryepondt S., Massey C., Edmonson P.D. (2016). 2nd Generation ODS FeCrAl Alloy Development for Accident-Tolerant Fuel Cladding.

[61] Qiao Y.J., Wang P., Qi W., Du S.Y., Liu Z., Meng F.P., Zhang X.H., Wang K., Li Q.W., Yao Z.D. (2020). Mechanism of Al on FeCrAl steam oxidation behavior and molecular dynamics simulations. J. Alloys Compd..

[62] Hofmann P. (1999). Current knowledge on core degradation phenomena, a review. J. Nucl. Mater..

[63] Isselin J., Kasada R., Kimura A. (2010). Corrosion behaviour of 16% Cr—4% Al and 16% Cr ODS ferritic steels under different metallurgical conditions in a supercritical water environment. Corros. Sci..

[64] Lee J.H., Kasada R., Kimura A., Okudab T., Inoue M., Ukai S., Ohnuki S., Fujisawa T., Abef F. (2011). Influence of alloy composition and temperature on corrosion behavior of ODS ferritic steels. J. Nucl. Mater..

[65] Ren J., Yu L.M., Liu Y.C., Ma Z.Q., Liu C.X., Li H.J., Wu J.F. (2019). Corrosion behavior of an Al added high-Cr ODS steel in supercritical water at 600 °C. Appl. Surf. Sci..

[66] Huttunen-Saarivirta E., Kuokkala V.T., Pohjanne P. (2014). Thermally grown oxide films and corrosion performance of ferritic stainless steels under simulated exhaust gas condensate conditions. Corros. Sci..

[67] Liu Y.C., Chen S.M., Ouyang F.Y., Kai J.J. (2018). Corrosion behavior of pre-oxidized HR—224 superalloy in supercritical water environment at 700 °C. J. Nucl. Mater..

[68] Terrani K.A., Pint B.A., Kim Y.J., Unocic K.A., Yang Y., Silva C.M., Meyer H.M., Rebak R.B. (2016). Uniform corrosion of FeCrAl alloys in LWR coolant environments. J. Nucl. Mater..

[69] Raiman S.S., Field K.G., Rebak R.B., Yamamoto Y., Terrani K.A. (2020). Hydrothermal corrosion of 2nd generation FeCrAl alloys for accident tolerant fuel cladding. J. Nucl. Mater..

[70] Hosemann P., Thau H.T., Johnson A.L., Maloy S.A., Li N. (2008). Corrosion of ODS steels in lead—Bismuth eutectic. J. Nucl. Mater..

[71] Takaya S., Furukawa T., Aoto K., Müller G., Weisenburger A., Heinzel A., Inoue M., Okudac T., Abed F., Ohnuki S. (2009). Corrosion behavior of Al—Alloying high Cr—ODS steels in lead-bismuth eutectic. J. Nucl. Mater..

[72] Unocic K.A., Pint B.A. (2014). Alloying and coating strategies for improved Pb—Li compatibility in DEMO—Type fusion reactors. J. Nucl. Mater..

[73] Unocic K.A., Hoelzer D.T. (2016). Evaluation of Pb—17Li compatibility of ODS Fe-12Cr-5Al alloys. J. Nucl. Mater..

[74] Klueh R.L., Shingledecker J.P., Swindeman R.W., Hoelzer D.T. (2005). Oxide dispersion-strengthened steels: A comparison of some commercial and experimental alloys. J. Nucl. Mater..

[75] Kasada R., Toda N., Yutani K., Cho H.S., Kishimoto H., Kimura A. (2007). Pre- and post-deformation microstructures of oxide dispersion strengthened ferritic steels. J. Nucl. Mater..

[76] Gong M., Zhou Z., Hu H., Zhang G., Li S., Wang M. (2015). Effects of Aluminum on Microstructure and Mechanical Behavior of 14Cr—ODS Steels. J. Nucl. Mater..

[77] Lee J.H. (2012). Development of oxide dispersion strengthened ferritic steels with and without aluminum. Front. Energy.

[78] Zhang G., Zhou Z., Mo K., Miao Y., Li S., Liu X., Wang M., Park J.S., Almer J., Stubbins J.F. (2016). The comparison of microstructures and mechanical properties between 14Cr—Al and 14Cr—Ti ferritic ODS alloys. Mater. Des..

[79] Gussev M.N., Field K.G., Yamamoto Y. (2017). Design, properties and weldability of advanced oxidation-resistant FeCrAl alloys. Mater. Des..

[80] Capdevila C., Miller M.K., Russell K.F. (2008). Aluminum partitioning during phase separation in Fe—20%Cr-6%Al ODS alloy. J. Mater. Sci..

[81] Vicente A.D.A., Moreno J.R.S., Espinosa D.C.R., Santos T.F.D.A., Tenorio J.A.S. (2019). Study of the high temperature oxidation and Kirkendall porosity in dissimilar welding joints between Fe—Cr—Al alloy and stainless steel AISI 310 after isothermal heat treatment at 1150 °C in air. J. Mater. Res. Technol..

[82] Wukusick C.S., Collis J.F. (1964). An Iron—Chromium—Aluminum Alloy Containing Yttrium. Mater. Res. Stand..

[83] Xu S., Zhou Z.J., Long F., Jia H.D., Guo N., Yao Z.W., Daymond M.R. (2019). Combination of back stress strengthening and Orowan strengthening in bimodal structured Fe—9Cr—Al ODS steel with high Al addition. Mater. Sci. Eng. A.

[84] Maji B.C., Ukai S., Oono N. (2021). Microstructural stability and intermetallic embrittlement in high Al containing FeCrAl—ODS alloys. Mater. Sci. Eng. A.

[85] Maréchal L., Lesage B., Huntz A.M., Molins R. (2003). Oxidation Behavior of ODS Fe—Cr—Al Alloys: Aluminum Depletion and Lifetime. Oxid. Met..

[86] Ukai S. (2012). Oxide dispersion strengthened steels. Comp. Nucl. Mater..

[87] Hsiung L.L., Fluss M.J., Tumey S.J., Choi B.W., Serruys Y., Willaime F., Kimura A. (2010). Formation mechanism and the role of nanoparticles in Fe—Cr ODS steels developed for radiation tolerance. Phys. Rev. B.

[88] Gelles D.S. (1994). Fusion materials. Semiannual Progress Report for Period Ending 31 March.

[89] Ukai S., Nishida T., Okuda T., Yoshitake T. (1998). R&D of oxide dispersion strengthened ferritic martensitic steels for FBR. J. Nucl. Mater..

[90] Lindau R., Möslang A., Rieth M., Klimiankou M., Materna-Morris E., Alamo A., Tavassoli A.A.F., Cayron C., Lancha A.M., Fernandez P. (2005). Present development status of EUROFER and ODS-EUROFER for application in blanket concepts. Fusion Eng. Des..

[91] Marriot J.B., Merz M., Nihoul J., Ward J. (1985). High Temperature Alloys: Their Exploitable Potential. Commission of the European Communities.

[92] Lu Z., Faulkner R.G., Riddle N., Martino F.D., Yang K. (2009). Effect of heat treatment on microstructure and hardness of Eurofer 97, Eurofer ODS and T92 steels. J. Nucl. Mater..

[93] Schaeublin R., Leguey T., Spätig P., Baluc N., Victoria M. (2002). Microstructure and mechanical properties of two ODS ferritic/martensitic steels. J. Nucl. Mater..

[94] Olier P., Bougault A., Alamob A., De Carlan Y. (2009). Effects of the forming processes and Y_2_O_3_ content on ODS—Eurofer mechanical properties. J. Nucl. Mater..

[95] Quadakkers W.J., Holzbrecher H., Briefs K.G., Beske H. (1989). Differences in growth mechanisms of oxide scales formed on ODS and conventional wrought alloys. Oxid. Met..

[96] Cueff R., Buscail H., Caudron E., Issartel C., Riffard F. (2003). Oxidation of alumina formers at 1173 K: Effect of yttrium ion implantation and yttrium alloying addition. Corros. Sci..

[97] Ul-Hamid A. (2003). Effect of Y_2_O_3_ Content on the Oxidation Behavior of Fe—Cr—Al—Based ODS Alloys. J. Mater. Eng. Perform..

[98] Ukai S., Sakamoto K., Ohtsuka S., Yamashita S., Kimura A. (2023). Review: Alloy design and characterization of a recrystallized FeCrAl-ODS cladding for accident-tolerant BWR fuels: An over-view of research activity in Japan. J. Nucl. Mater..

[99] Ukai S., Harada M., Okada H., Inoue M., Nomura S., Shikakura S., Asabe K., Nishida T., Fujiwara M. (1993). Alloying design of oxide dispersion strengthened ferritic steel for long life FBRs core materials. J. Nucl. Mater..

[100] Ukai S., Nishida T., Okada H., Okuda T., Fujiwara M., Asabe K. (1997). Development of Oxide Dispersion Strengthened Ferritic Steels for FBR Core Application, (I). Improvement of Mechanical Properties by Recrystallization Processing. J. Nucl. Sci. Technol..

[101] Ukai S., Fujiwara M. (2002). Perspective of ODS alloys application in nuclear environments. J. Nucl. Mater..

[102] Yamashita S., Ohtsuka S., Akasaka N., Ukai S., Ohnuki S. (2004). Formation of nanoscale complex oxide particles in mechanically alloyed ferritic steel. Philos. Mag. Lett..

[103] Miller M.K., Hoelzer D.T., Kenik E.A., Russell K.F. (2004). Nanometer scale precipitation in ferritic MA/ODS alloy MA957. J. Nucl. Mater..

[104] Klimiankou M., Lindau R., Möslang A. (2003). HRTEM Study of yttrium oxide particles in ODS steels for fusion reactor application. J. Cryst. Growth.

[105] Lescoat M.L., Ribis J., Gentils A., Kaïtasov O., De Carlan Y., Legris A. (2012). In situ TEM study of the stability of nano-oxides in ODS steels under ion-irradiation. J. Nucl. Mater..

[106] Czyrska-Filemonowicz A., Szot K., Wasilkowska A., Gil A., Quadakkers W.J. (1999). Microscopy (AFM, TEM, SEM) studies of oxide scale formation on FeCrAI based ODS alloys. Solid State Ion..

[107] Klimiankou M., Lindau R., Möslang A. (2005). Energy-filtered TEM imaging and EELS study of ODS particles and Argon-filled cavities in ferritic-martensitic steels. Micron.

[108] Klimiankou M., Lindau R., Moslang A. (2004). TEM characterization of structure and composition of nanosized ODS particles in reduced activation ferritic-martensitic steels. J. Nucl. Mater..

[109] Lu C.Y., Lu Z., Wang X., Xie R., Li Z.Y., Higgins M., Liu C.M., Gao F., Wang L.M. (2017). Enhanced Radiation-tolerant Oxide Dispersion Strengthened Steel and its Microstructure Evolution under Helium-implantation and Heavy-ion Irradiation. Sci. Rep..

[110] Klueh R.L., Maziasz P.J., Kim I.S., Heatherly L., Hoelzer D.T., Hashimoto N., Kenik E.A., Miyahara K. (2002). Tensile and creep properties of an oxide dispersion-strengthened ferritic steel. J. Nucl. Mater..

[111] Sakasegawa H., Legendre F., Boulanger L., Brocq M., Chaffron L., Cozzika T., Malaplate J., Henry J., De Carlan Y. (2011). Stability of non-stoichiometric clusters in the MA957 ODS ferrtic alloy. J. Nucl. Mater..

[112] Ribis J., De Carlan Y. (2012). Interfacial strained structure and orientation relationships of the nanosized oxide particles deduced from elasticity-driven morphology in oxide dispersion strengthened materials. Acta Mater..

[113] Kishimoto H., Kasad R., Hashitomi O., Kimura A. (2009). Stability of Y-Ti complex oxides in Fe-16Cr-0.1Ti ODS ferritic steel before and after heavy-ion irradiation. J. Nucl. Mater..

[114] Zhang B.Q., Lu L., Lai M.O. (2003). Evolution of vacancy densities in powder particles during mechanical milling. Phys. B Condens. Matter..

[115] Xu J., Liu C.T., Miller M.K., Chen H.M. (2009). Nanocluster-associated vacancies in nanocluster-strengthened ferritic steel as seen via positron-lifetime spectroscopy. Phys. Rev. B.

[116] Alinger M.J. (2009). Positron annihilation characterization of nanostructured ferritic alloys. Mater. Sci. Eng. A.

[117] Krsjak V., Szaraz Z., Hahner P. (2012). Positron annihilation lifetime study of oxide dispersion strengthened steels. J. Nucl. Mater..

[118] Fu C.L., Krcmar M., Painter G.S., Chen X.Q. (2007). Vacancy mechanism of high oxygen solubility and nucleation of stable oxygen-enriched clusters in Fe. Phys. Rev. Lett..

[119] Jiang Y., Smith J.R., Odette G.R. (2009). Formation of Y-Ti-O nanoclusters in nano- structured ferritic alloys: A first-principles study. Phys. Rev. B.

[120] Zhang Z.W., Yao L., Wang X.L., Miller M.K. (2015). Vacancy-controlled ultrastable nanoclusters in nano-structured ferritic alloys. Sci. Rep..

[121] Vallinayagam M., Posselt M., Faßbender J. (2019). Investigation of structural models for O–Y and O–Y–Ti clusters in bcc Fe: A density functional theory study. J. Phys. Condens. Matter.

[122] Barnard L., Cunningham N., Odette G.R., Szlufarska I., Morgan D. (2015). Thermodynamic and kinetic modeling of oxide precipitation in nanostructured ferritic alloys. Acta Mater..

[123] Boulnat X., Perez M., Fabregue D., Cazottes S., De Carlan Y. (2016). Characterization and modeling of oxides precipitation in ferritic steels during fast non-isothermal consolidation. Acta Mater..

[124] Rogozhkina S.V., Bogacheva A.A., Kirillova D.I., Nikitina A.A., Orlova N.N., Aleeva A.A., Zaluzhnyia A.G., Kozodaev M.A. (2014). Effect of Alloying with Titanium on the Microstructure of an Oxide Dispersion Strengthened 13.5% Cr Steel. Phys. Met. Metallogr..

[125] Oksiuta Z., Baluc N. (2009). Optimization of the chemical composition and manufacturing route for ODS RAF steels for fusion reactor application. Nucl. Fusion.

[126] Massey C.P., Hoelzer D.T., Seibert R.L., Edmondson P.D., Kini A., Gault B., Terrani K.A., Zinkle S.J. (2019). Microstructural evaluation of a Fe-12Cr nanostructured ferritic alloy designed for impurity sequestration. J. Nucl. Mater..

[127] Capdevila C., Pimentel G., Aranda M.M., Rementeria R., Dawson K., Urones-Garrote E., Tatlock G.J., Miller M.K. (2015). Role of Y-Al Oxides During Extended Recovery Process of a Ferritic ODS Alloy. JOM.

[128] Zhang C.H., Kimura A., Kasada R., Jang J., Kishimoto H., Yang Y.T. (2011). Characterization of the oxide particles in Al-added high-Cr ODS ferritic steels. J. Nucl. Mater..

[129] Unocic K.A., Pint B.A., Hoelzer D.T. (2016). Advanced TEM characterization of oxide nanoparticles in ODS Fe-12Cr-5Al alloys. J. Mater. Sci..

[130] Hsiung L.L., Fluss M.J., Kimura A. (2010). Structure of oxide nanoparticles in Fe–16Cr MA/ODS ferritic steel. Mater. Lett..

[131] Dubiel B., Osuch W., Wro´bel M., Ennis P.J., Czyrsk-Filemonowicz A. (1995). Correlation of the microstructure and the tensile deformation of incology MA956. J. Mater. Process. Technol..

[132] Czyrska-Filemonowicz A., Clemens D., Quadakkers W.J. (1995). The effect of high temperature exposure on the structure and oxidation behaviour of mechanically alloyed ferritic ODS alloys. J. Mater. Process. Technol..

[133] Chen C.L., Richter A., Kögler R., Talut G. (2011). Dual beam irradiation of nanostructured FeCrAl oxide dispersion strengthened steel. J. Nucl. Mater..

[134] Chinnappan R. (2014). Thermodynamic stability of oxide phases of Fe-Cr based ODS steels via quantum mechanical calculations. CALPHAD Comput. Coupling Phase Diagr. Thermochem..

[135] Kamikawa R., Ukai S., Kasai S., Oono N., Zhang S., Sugino Y., Masuda H., Sato E. (2018). Cooperative grain boundary sliding in creep deformation of FeCrAl—ODS steels at high temperature and low strain rate. J. Nucl. Mater..

[136] Mohan S., Kaur G., Panigrahi B.K., David C., Amarendra G. (2018). Effect of Zr and Al addition on nanocluster formation in oxide dispersion strengthened steel—An ab initio study. J. Alloys Compd..

[137] Qian Q., Wang Y.R., Jiang Y., He C., Hu T. (2019). Nucleation of Y-X-O (X=Al, Ti, or Zr) NCs in nano-structured ferritic alloys: A first principles comparative study. J. Nucl. Mater..

[138] Dou P., Kimura A., Kasada R., Okuda T., Inoue M., Ukai S., Ohnukid S., Fujisawa T., Abe F. (2014). TEM and HRTEM study of oxide particles in an Al—Alloyed high—Cr oxide dispersion strengthened steel with Zr addition. J. Nucl. Mater..

[139] Takaya S., Furukawa T., Inoue M., Fujisawa T., Okudac T., Abed F., Ohnuki S., Kimura A. (2010). Corrosion resistance of Al-alloying high Cr-ODS steels in stagnant lead-bismuth. J. Nucl. Mater..

[140] Takaya S., Furukawa T., Müller G., Heinzel A., Jianu A., Weisenburger A., Aoto K., Inoue M., Okuda T., Abe F. (2012). Al—Containing ODS steels with improved corrosion resistance to liquid lead- bismuth. J. Nucl. Mater..

[141] Yu C.Z., Oka H., Hashimoto N., Graduate S.O. (2011). Development of damage structure in 16Cr-4Al ODS steels during electron-irradiation. J. Nucl. Mater..

[142] García-Junceda A., García-Rodríguez N., Campos M., Cartón-Cordero M., Torralba J.M. (2015). Effect of Zirconium on the Microstructure and Mechanical Properties of an Al—Alloyed ODS Steel Consolidated by FAHP. J. Am. Ceram. Soc..

[143] Wang X., Lu Z., Li Z.Y., Shi Y.N., Xu H.J. (2022). Effect of Zr content on the microstructure and hardness of 12Cr—5Al ODS FeCrAl alloy consolidated by spark plasma sintering. Mater. Charact..

[144] Gao R., Zhang T., Wang X.P., Fang Q.F., Liu C.S. (2014). Effect of zirconium addition on the microstructure and mechanical properties of ODS ferritic steels containing aluminum. J. Nucl. Mater..

[145] Dou P., Sang W., Kimura A. (2019). Morphology, crystal and metal/oxide interface structures of nanoparticles in Fe-15Cr-2W-0.5Ti-7Al-0.4Zr-0.5Y_2_O_3_ ODS steel. J. Nucl. Mater..

[146] Ren J., Yu L.M., Liu Y.C., Liu C.X., Li H.J., Wu J.G. (2018). Effects of Zr Addition on Strengthening Mechanisms of Al-Alloyed High-Cr ODS Steels. Materials.

[147] Ramar A., Oksiuta Z., Baluc N., Schäublin R. (2007). Effect of mechanical alloying on the mechanical and microstructural properties of ODS Eurofer 97. Fusion Eng. Des..

[148] Dou P., Jiang S.M., Qiu L.L., Kimura A. (2020). Effects of contents of Al, Zr and Ti on oxide particles in Fe-15Cr-2W-0.35Y_2_O_3_ ODS steels. J. Nucl. Mater..

[149] Wu S.J., Li J., Li W.H., Liu S. (2020). Characterization of oxide dispersoids and mechanical properties of 14Cr-ODS FeCrAl alloys. J. Alloys Compd..

[150] Massey C.P., Dryepondt S.N., Edmondson P.D., Terrani K.A., Zinkle S.J. (2018). Influence of mechanical alloying and extrusion conditions on the microstructure and tensile properties of Low-Cr ODS FeCrAl alloys. J. Nucl. Mater..

[151] Massey C.P., Edmondson P.D., Unocic K.A., Yang Y., Dryepondt S.N., Kini A., Gault B., Terrani K.A., Zinkle S.J. (2020). The effect of Zr on precipitation in oxide dispersion strengthened FeCrAl alloys. J. Nucl. Mater..

[152] Massey C.P., Dryepondt S.N., Edmondson P.D., Frith M.G., Littrell K.C., Kini A., Gault B., Terrani K.A., Zinkle S.J. (2019). Multiscale investigations of nanoprecipitate nucleation, growth, and coarsening in annealed low-Cr oxide dispersion strengthened FeCrAl powder. Acta Mater..

[153] Shi Y.N., Lu Z., Yu L., Xie R., Ren Y.H., Yang G. (2020). Microstructure and tensile properties of Zr-containing ODS-FeCrAl alloy fabricated by laser additive manufacturing. Mater. Sci. Eng. A.

[154] Klimiankou M., Lindau R., Moslang A., Schroder J. (2005). TEM study of PM2000 steel. Powder Metall..

[155] Klimenkou M., Moslang A., Lindau R. (2008). EELS analysis of complex precipitates in PM2000 steel. Eur. Phys. J.-Appl. Phys..

[156] Zhou X.S., Ma Z.Q., Yu L.M., Huang Y., Li H.J., Liu Y.C. (2019). Formation mechanisms of Y-Al-O complex oxides in 9Cr-ODS steels with Al addition. J. Mater. Sci..

[157] Marquis E.A. (2008). Core/shell structures of oxygen-rich nanofeatures in oxide-dispersion strengthened Fe-Cr alloys. Appl. Phys. Lett..

[158] London A.J., Lozano-Perez S., Moody M.P., Amirthapandian S., Panigrahi B.K., Sundar C.S., Grovenor C.R. (2015). Quantification of oxide particle composition in model oxide dispersion strengthened steel alloys. Ultramicroscopy.

[159] Badjeck V., Walls M.G., Chaffron L., Malaplate J., March K. (2015). New insights into the chemical structure of Y_2_Ti_2_O_7_ nanoparticles in oxide dispersion-strengthened steels designed for sodium fast reactors by electron energy- loss spectroscopy. J. Nucl. Mater..

[160] Higgins M.P., Liu C.Y., Lu Z., Shao L., Wang L.M., Gao F. (2016). Crossover from disordered to core-shell structures of nano-oxide Y_2_O_3_ dispersed particles in Fe. Appl. Phys. Lett..

[161] Xu H.J., Lu Z., Ukai S., Oono N., Liu C.M. (2017). Effects of annealing temperature on nanoscale particles in oxide dispersion strengthened Fe-15Cr alloy powders with Ti and Zr additions. J. Alloys Comp..

[162] Williams C.A., Marquis E.A., Cerezo A., Smith G.D. (2010). Nanoscale characterisation of ODS-Eurofer 97 steel: An atom-probe tomography study. J. Nucl. Mater..

[163] Murali D., Panigrahi B.K., Valsakumar M.C., Chandra S., Sundar C.S., Raj B. (2010). The role of minor alloying elements on the stability and dispersion of yttria nanoclusters in nanostructured ferritic alloys: An ab initio study. J. Nucl. Mater..

[164] Oono H., Ukai S., Hayashi S., Ohtsuka S., Kaito T., Kimura A., Torimaru T., Sakamoto K. (2017). Growth of oxide particles in FeCrAl-oxide dispersion strengthened steels at high temperature. J. Nucl. Mater..

[165] Klueh R.L. (1982). Chromium-molybdenum steels for fusion reactor first walls: A review. Nucl. Eng. Des..

[166] Klueh R.L., Bloom E.E. (1985). The development of ferritic steels for fast induced—Radioactivity decay for fusion reactor applications. Nucl. Eng. Des. Fusion.

[167] Kimura H., Kayano T., Misawa H. (1994). Designation of alloy composition of reduced-activation martensitic steel. J. Nucl. Mater..

[168] Ukai S., Harada M., Okada H., Inoue M., Nomura S., Shikakura S., Nishida T., Fujiwara M., Asabe K. (1993). Tube manufacturing and mechanical properties of oxide dispersion strengthened ferritic steel. J. Nucl. Mater..

[169] Ukai S., Yoshitake T., Mizuta S., Matsudaira Y., Hagi S., Kobayashi T. (1999). Preliminary Tube Manufacturing of Oxide Dispersion Strengthened Ferritic Steels with Recrystallized Structure. J. Nucl. Sci. Technol..

[170] Miller M.K., Kenik E.A., Russell K.F., Heatherly L., Hoelzer D.T., Maziasz P.J. (2003). Atom probe tomography of nanoscale particles in ODS ferritic alloys. Mater. Sci. Eng. A.

[171] Dou P., Kimura A., Kasada R.Y., Okuda T., Inoue M., Ukai S., Ohnukid S., Fujisawa T., Abe F. (2013). Effects of titanium concentration and tungsten addition on the nano-mesoscopic structure of high-Cr oxide dispersion strengthened (ODS) ferritic steels. J. Nucl. Mater..

[172] El-Dasher B., Farmer J., Ferreira J., Caro M.S., Rubenchik A., Kimura A. (2011). Corrosion of oxide dispersion strengthened iron—Chromium steels and tantalum in fluoride salt coolant: An in situ compatibility study for fusion and fusion—Fission hybrid reactor concepts. J. Nucl. Mater..

[173] Sakasegawa H., Hirose T., Suzuki T., Kohyama A., Katoh Y., Harada T., Asakura K., Kumagai T. (2001). Report of IEA Workshop on Reduced—Activation Ferritic/Martensitic Steels.

